# Combining sorafenib with spermine and sphingosine synergistically enhances anticancer efficacy by modulating metabolic pathways and gut microbiome in hepatocellular carcinoma

**DOI:** 10.7150/ijbs.118753

**Published:** 2026-01-01

**Authors:** Hay-Ran Jang, Hyun-Jin Kim, Bo-Young Kim, Jae-Hoon Jeong, Jeon-Kyung Kim, Jin Ah Won, Hye Hyun Yoo, Yong Gu Lee, Hyungshin Yim

**Affiliations:** 1 Department of Pharmacy, College of Pharmacy, Hanyang University, Ansan, Republic of Korea.; 2 School of Pharmacy, Institute of New Drug Development, Jeonbuk National University, Jeonju, Republic of Korea.; 3 Pharmacomicrobiomics Research Center, College of Pharmacy, Hanyang University, Ansan, Republic of Korea.

**Keywords:** spermine, sphingosine, microbiome metabolite, sorafenib, liver cancer

## Abstract

Hepatocellular carcinoma (HCC), which makes up about 90% of liver cancer, is the third leading cause of cancer-related death. Recent studies suggest that metabolites derived from the gut microbiome may offer new therapeutic opportunities for HCC. In this study, we explored whether microbial metabolites could enhance the effectiveness of sorafenib, a first-line multi-kinase inhibitor used in advanced HCC. Through a screen of a microbiome metabolite library, we identified spermine and sphingosine as potential candidates that boosted anticancer effects of sorafenib in HepG2, Huh7, and SK-Hep-1 cells. These metabolites worked synergistically with sorafenib to suppress tumor growth in cultured HCC cells, patients-derived HCC organoids, and a xenograft mouse model. Mechanistically, spermine triggered cell cycle arrest at the S phase, while sphingosine and sorafenib induced G1 arrest, contributing to an increased sub-G1 population and apoptosis when combined. Notably, sorafenib treatment led to the downregulation of *SMOX* (a key catabolic enzyme for spermine), as well as *SPHK1* and* CERS1* (critical enzymes involved in sphingosine metabolism), whose high expression levels are associated with poorer survival outcomes in liver cancer patients according to TCGA data. A 16S rRNA sequencing analysis revealed that combination of sorafenib with spermine or sphingosine alters the gut microbiome, increasing the relative abundance of *Faecalibaculum,* inversely correlated with tumor sizes in a xenograft mouse model. Therefore, we propose that combining sorafenib with spermine or sphingosine could enhance its anti-HCC effects by promoting apoptosis and reducing the expression of metabolic enzymes. Moreover, *Faecalibaculum* may serve as a potential microbiome-based prognostic marker for HCC.

## 1. Introduction

Liver cancer is the third most common cause of cancer-related death worldwide in 2022 accounting for 7.8% of all cancer deaths 1. Hepatocellular carcinoma (HCC) is the most common liver cancer accounting for approximately 90% 2. It usually develops with chronic liver disease and requires a complex, multidisciplinary treatment, because its development is driven by various primary carcinogens, including hepatitis B or C virus, metabolic disorders, alcohol consumption, and gene mutation 3-5. In systemic chemotherapy, sorafenib, a multi-kinase inhibitor targeting VEGFR, PDGFR, and RAF/MAPK, is the standard first-line treatment for HCC patients 3-5. Another multi-kinase inhibitor, lenvatinib, has demonstrated non-inferiority to sorafenib and is also approved as a first-line option for treating advanced HCC 6. For patients who progress on sorafenib, second-line treatment options include regorafenib and cabozantinib 7. Recently, immunotherapy has gained prominence in HCC treatment, particularly with the use of atezolizumab, an anti-PD-L1 immune checkpoint inhibitor 8. Especially when atezolizumab combined with bevacizumab has shown 5.8 months longer median overall survival compared to sorafenib in unresectable HCC (NCT03434379) 8, 9. However, atezolizumab combined with bevacizumab causes severe adverse effects including as proteinuria, hypertension, and fatigue 9 and remains limited in the patients with a history of autoimmune disease 10 or varicose vein 11. Therefore, sorafenib is still a basis of treatment for advanced HCC with a proven survival benefit, multi-targets including RAS, VEGFR, and PDGFR, a manageable safety profile, and potential for combination therapy 4, 12. On the other hand, sorafenib has its own limitations, including a relatively modest survival benefit in advanced HCC 13. Due to the heterogeneity of HCC, patients may exhibit primary resistance to sorafenib depending on their genetic background 14. In contrast, acquired resistance to sorafenib is often associated with the activation of pathways such as PI3K/AKT/mTOR 15 and MAPK/ERK pathway 16. To overcome this resistance, combination therapies targeting these pathways such as the use of Torin2 17 or USP22 shRNA 18, have been explored to enhance the therapeutic response to sorafenib.

Emerging evidence for an important contribution of the gut microbiome to carcinogenesis suggests that the gut microbiome plays a crucial role in HCC progression through dysbiosis, bacteria metabolites, and immunosuppression via a leaky gut 19, 20. In the patients with advanced HCC, the gut microbiome is characterized by an increase in potentially pathologic bacteria and a decrease with beneficial ones. When dysbiosis of the gut microbiome occurs, certain microbiomes-related molecules or metabolites could trigger inflammation, suppress immune function, and cause liver toxicity, which may promote cancer development 19, 20. In elderly patients with advanced HCC, the gut microbiome tends to shift, with an increase in harmful bacteria including *Shigella* and *Veillonella* and a decrease in beneficial ones such as *Bifidobacterium* 20. Thus, the composition of beneficial bacteria and microbiome-derived metabolites would have translational potential for HCC therapy. Bacteria such as *Akkermansia muciniphila* and *Faecalibacterium prausnitzii* have been associated with better responses to immunotherapies such as anti-PD-1 treatment, by promoting T-cell activation and reducing immune suppression within the tumor microenvironment 21, 22. Preclinical studies also reported the importance of microbiome-derived metabolites in immune checkpoint blockade, through the interaction with the host 23, 24. Polyamines including putrescine and spermidine produced by lactic acid bacteria, have been shown to exert colonic epithelial proliferation and macrophage differentiation 25. Therefore, microbiome-derived metabolites can be applicable for both in clinics and translational research for HCC.

In this study, to improve the relatively modest survival benefit of sorafenib in advanced HCC, we investigated microbial metabolites that could enhance sorafenib's efficacy. By screening a microbiome metabolite library, spermine and sphingosine were identified as potential enhancers of sorafenib's anticancer effects in HCC cell lines that HepG2 (derived from hepatoblastoma and representing well-differentiated HCC, low tumorigenicity), Huh7 (a moderately differentiated HCC, moderate tumorigenicity), and SK-Hep-1 (metastatic HCC, high tumorigenicity) 26, 27. We found that spermine and sphingosine themselves exhibited anti-proliferating activities in HCC cells through cell cycle arrest in the S phase and G1 phase, respectively. In addition, sorafenib treatment led to the downregulation of *SMOX* (a key catabolic factor for spermine), as well as *SPHK1* and* CERS1* (factors involved in sphingosine metabolism). Notably, high expression levels of *SMOX, SPHK1,* and* CERS1* were inversely associated with the survival rates in 364 liver cancer patients based on TCGA data. Moreover, microbiome profiling in mice treated with sorafenib in combination with spermine or sphingosine showed a negative correlation between tumor size and the relative abundance of *Faecalibaculum*, suggesting its potential as a microbiome-based biomarker for HCC.

## 2. Materials and Methods

### 2.1. Materials

For cell culture, Dulbecco's modified Eagle's medium (DMEM), RPMI-1640 medium, fetal bovine serum (FBS), penicillin, and streptomycin were used (Corning Cellgro, Manassas, VA, USA). Sorafenib, spermine, D-erythro-sphingosine (sphingosine), and a gut microbial metabolite library (Table S1) were obtained from Med Chem Express (Princeton, NJ, USA). For LC/MS-MS analysis, terfenadine (used as internal standard, IS) and heptafluorobutyric acid (HFBA) were purchased from Sigma-Aldrich (Steinheim, Germany). Formic acid (FA) was purchased from Supelco (Merck KGaA, Darmstadt, Germany). HPLC grade methanol (MeOH) was obtained from J.T. Baker (Phillipsburg, NJ). Distilled water (DW) was prepared using a Milli-Q purification system (Millipore, Bedford, MA).

### 2.2. Cell culture

Human hepatocellular carcinoma Huh7, SK-Hep-1, and HepG2 cells were purchased from KCLB (Seoul, Korea). The cells were verified by STR profiling and screened for mycoplasma contamination. Huh7 and HepG2 were grown in RPMI 1640 and SK-Hep-1cells were in DMEM with 10% FBS and antibiotics in a 5% CO_2_ incubator at 37 °C.

### 2.3. Cell viability assay

MTT (3-(4, 5-dimethylthiazol-2-yl)- 2, 5-diphenyltetrazolium bromide) was obtained from Sigma-Aldrich (Burlington, MA, USA) and used for cell viability assay. According to the manufacturer's protocol and a previous study 28, 2×10^4^ cells/ml of HepG2, Huh7, or SK-Hep-1 cells were placed in a 96 well-plate and treated with sorafenib, spermine, or sphingosine for 48 h. Next, the cells were treated with 2.5 mg/ml of MTT and incubated at 37 °C for 2 h. The intensity of formazan dye was then measured with a microplate reader (SpectraMax M4, Molecular Devices; Sunnyvale, CA, USA) at an absorbance of 540 nm. In combination experiments, the combination index (CI) was calculated from CI equation algorithms and displayed using Compusyn software (ComboSyn Inc; Paramus, NJ, USA) and SynergyFinder (https://synergyfinder.org): CI < 1, synergism; CI =1, additive effect; and CI > 1 antagonism.

### 2.4. Fluorometric caspase-3 activity assay

Cell lysates (30 μg) were treated with 200 nM Ac-DEVD-AMC (Med Chem Express; Princeton, NJ, USA) in reaction buffer (2 mM DTT, 20 mM HEPES [pH 7.5], and 10% glycerol) at 37 °C, in accordance with a previous study 29. The reaction was monitored with a SpectraMax M4 microplate reader (Molecular Devices; Sunnyvale, CA, USA) by observing fluorescence emissions at 430 nm (excitation at 360 nm).

### 2.5. Immunofluorescence

Immunofluorescence was performed in accordance with a previous study 28, 30. Briefly, SK-Hep-1 cells grown on coverslips were fixed with 4% paraformaldehyde. Methanol was used for permeabilization. The cells were washed three times with PBS containing 0.1% Triton X-100 (PBST), incubated overnight at 4 °C in PBST and 3% bovine serum albumin to block nonspecific reactions, and then incubated with anti-cleaved caspase-3 (Cell Signaling, 9661S; Danvers, MA, USA) and anti-α-tubulin (Sigma-Aldrich, T6074) antibodies. The cells were then washed three times with PBST and incubated with FITC-conjugated anti-rabbit secondary antibodies (Jackson ImmunoResearch Laboratories, West Grove, PA, USA), Cy™3-conjugated anti-mouse secondary antibodies (Jackson ImmunoResearch Laboratories). DAPI (4′, 6- diamidine-2-phenylindole) (Sigma-Aldrich) was used for staining nuclear DNA. Images of cells were collected and evaluated with an FW3000 confocal microscope (Olympus; Tokyo, Japan).

### 2.6. Fluorescence-activated cell sorting (FACS) analysis

To validate the population of cells in each phase of the cell cycle, FACS analysis was performed according to the previous reports 28, 31. Cells were treated with trypsin, collected, and fixed in 75% ethanol. Then cells were stained with propidium iodide solution at the concentration of 30 µg/ml, and subjected to a FACS analysis. Cells were sorted by a Guava easyCyte^TM^ flow cytometry machine (Millipore; Billerica, MA, USA). The data were analyzed with Incyte™ software (Millipore).

### 2.7. LC/MS-MS analysis

Samples for LC/MS-MS analysis were prepared and analyzed as previously described with a slight modification 32. Briefly, normalized cell lysate samples were prepared in 1.5 ml Eppendorf tubes and spiked with internal standard (IS; 100 μl, 200 ng/ml in 0.2% (v/v) FA in MeOH). The mixtures were vortexed for 1 minute and centrifuged at 12,000 rpm for 5 minutes at 4 °C. The supernatants (20 μl) were further diluted with IS solution (80 μl, 8.3 ng/ml in 0.2% (v/v) FA in MeOH). The resulting solutions were transferred to analytical vials and subjected to HPLC-MS/MS. Spermine was quantified using an Agilent 1290 Infinity UPLC system coupled to an Agilent 6470 triple-quadrupole mass spectrometer (Agilent Technologies, Santa Clara, CA, USA). Chromatographic separation was performed on a Waters Acquity UPLC® C18 column (2.1 × 100 mm, 1.7 μm) at 40°C. The mobile phases were 0.1% (v/v) FA with 1.5 mM HFBA in distilled water (solvent A) and 1.5 mM HFBA in MeOH (solvent B). The flow rate was 0.3 ml/min and the injection volume was 2 μl. Mass spectrometry was performed using electrospray ionization in positive ion mode with multiple reaction monitoring (MRM) detection. The MRM transitions were m/z 203.0 → 112.0 for spermine and m/z 472.0 → 436.0 for terfenadine (IS). Calibration curve for the quantification of spermine was constructed over the concentration range of 10-500 ng/ml.

### 2.8. Immunoblot analysis

SK-Hep-1 cells were lysed in buffer [2 mM MgCl_2_, 1 mM EGTA, 1 mM dithiothreitol (DTT), 50 mM β-glycerophosphate, 25 mM NaF, 1 mM Na vanadate, 0.5% Triton X-100, 20 mM Tris (pH 7.5), 100 mg/ml PMSF, and protease inhibitor cocktail (Roche; Indianapolis, IN, USA)] for 1 hours on ice. Lysates were centrifuged to collect the supernatants. Protein concentrations were adjusted, and samples were resolved by SDS-PAGE, followed by immunoblotting with the following primary antibodies: cyclin A (sc-751, Santa Cruz Biotechnology, Dallas, TX, USA, 1:1000), cyclin D1 (sc-753, Santa Cruz Biotechnology, 1:1000), cyclin E1 (sc-481, Santa Cruz Biotechnology, 1:1000), p21^WAF1/CIP1^ (sc-6246, Santa Cruz Biotechnology, 1:250), β-actin (sc-47778, Santa Cruz Biotechnology, 1:1000). Bands were visualized using an Odyssey infrared imaging system (LI-COR Biosciences; Lincoln, NE, USA) and band intensities were quantified using LI-COR Odyssey software.

### 2.9. *Ex vivo* hepatocellular carcinoma organoids models

Patient-derived HCC SNU-423-CO organoids 33 were obtained from KCLB (Seoul, Korea) and maintained using the human HCC organoid culture kit (Med Chem Express, Princeton, NJ, USA), supplemented with 50 ng/ml EGF, 100 ng/ml FGF, 25 ng/ml HGF, 10 mM forskolin, 1 × B27, 10 mM nicotinamide, 5 mM A83-01, 1.25 mM N-acetylcysteine, and 50 mg/ml primocin 34. According to the manufacturer's protocol, organoids were embedded in basement membrane matrix (Thermo Fisher Scientific; Wilmington, DE, USA) and seeded into 24- or 96-well plates. After polymerization, organoid culture medium (Med Chem Express, Princeton, NJ, USA) was added and replaced every 3-4 days. Organoids were passaged every 1-2 weeks using TrypLE Express solution (Thermo Fisher Scientific). Cultures were maintained at 37 °C in a humidified 5% CO_2_ incubator. For cell viability assays, organoids were seeded in a 5×10^3^ cells /10 μl of basement membrane matrix droplets in 96-well plates and treated with culture medium containing the indicated reagents. Organoid formation efficiency was assessed by CellTiter-Glo 3D reagent (Promega, G9681; USA) according to the manufacturer's protocols.

### 2.10. Animal studies with xenograft mouse model

Six-week-old, BALB/c male nude mice (Orient Bio, Seoul, Korea) were subcutaneously injected in the upper left thigh with a mixture of SK-Hep-1 cells and 50% Matrigel at a 1:1 ratio (1 ×10^7^ cells/100 μl PBS/mouse). When tumor sizes were measured one week after injection, the tumor volumes ranged from 60 to 80 mm³. Thirty mice were randomly divided into six groups (five mice per group) and assigned to receive vehicle, sorafenib, spermine, sphingosine, sorafenib plus spermine, or sorafenib plus Sphingosine. The tumor volumes were measured twice a week and calculated using the formula: (length [mm] x width^2^ [mm^2^]/2, width < length). Sorafenib (30 mg/kg, orally), spermine (10 mg/kg, intraperitoneal), and sphingosine (25 mg/kg, intraperitoneal) were administered to the assigned groups three times a week for 32 days. After the final drug administration, the mice were sacrificed, and the tumors were removed and weighed. All animal experiments were approved and managed according to guidelines from the Institutional Animal Care and Use Committee, Hanyang University (HY-IACUC-2023-0309A).

### 2.11. Quantitative reverse transcription polymerase chain reaction (qRT-PCR)

To extract total RNA, cells or tissues were treated with TRIzol reagent (Molecular Research Center, #TR118; Cincinnati, OH, USA). Total RNA was quantified on a NanoDrop (Thermo Fisher Scientific; Wilmington, DE, USA) according to the manufacturers' directions and previous studies 35, 36. To synthesize cDNA, a First Strand cDNA synthesis kit (Thermo Fisher Scientific) was used. The generated cDNA was mixed with SYBR green master mix (Meridian Bioscience Inc., Cincinnati, OH, USA) and gene-specific primers (Table S2). qRT-PCR was performed using a CFX96 real-time PCR system (Bio-Rad Laboratories, Hercules, CA, USA).

### 2.12. Lentivirus-based shRNA preparation

To deplete *SMOX*,* SPHK1*, and* CERS1*, we designed lentivirus-based shRNA transfer plasmids to target human *SMOX* (gene access no. NM_175841) at positions 574-594 (5'-AGGACGTGGTTGAGGAATTCA-3', shSMOX), human *SPHK1* (gene access no. NM_001142602) at positions 1126-1146 (5'-AGGGCCCGGTAGATGCACACC-3', shSPHK1), and human *CERS1* (gene access no. NM_001387443) at positions 526-549 (5'-ACATTGCAGCCGCCTACCTGC-3', shCERS1). The lentiviruses were generated according to the previous report 37. The infected cells were selected using 2 µg/ml puromycin for 2 days. Depleted cells were treated with sorafenib, spermine, and/or sphingosine at the indicated concentration.

### 2.13. Bioinformatics analysis

Patient data for liver cancer were extracted from TCGA (https://cancergenome.nih.gov/) with OS available for 364 patients of an online database (www.kmplot.com), which includes gene expression profiles and survival information, in accordance with previous reports 35, 38. Patient expression values for* SMOX*, *SPHK1*, *CERS1* (other names *LASS1, GDF1*), *SMS*, *CER1*, and *SGPP1* were extracted and used for the survival analysis after excluding biased arrays. The samples were split into groups with high and low expression of *SMOX*, *SPHK1*, *CERS1*, *SMS*, *CER1*, and *SGPP1*. The calculations were performed using an R script. A *p-*value of less than 0.05 was defined as statistically significant. The log rank *P* and hazard ratios (HRs) with 95% confidence intervals were calculated according to the formulas on the database's webpage (Table S4). Geneset GSE96794 provides the transcriptomic profile of Huh7 cells after 24 hours of sorafenib treatment. For normalization, Log2-fold changes in the RNA-Seq dataset were multiplied by 2 and subsequently converted to gene symbols using the platform annotation provided in the database.

### 2.14. Gut microbiome profiling and analysis

To analyze gut microbiome, cecal samples were collected from the mice and immediately preserved in DNA/RNA Shield solution (Zymo Research, Irvine, CA, USA), then stored at -80 °C until further processing. DNA was extracted using the QIAGEN stool prep kit (Germany) according to the manufacturer's instructions. The V4 region of the bacterial 16S rRNA gene was amplified using barcoded primers: forward 517F (5′-GCCAGCMGCCGCGGTAA-3′) and reverse 806R (5′-GGACTACHVGGGTWTCTAAT-3′). The primers included degenerate nucleotides according to the IUPAC code: M (A/C), H (A/C/T), V (A/C/G), and W (A/T). To generate 301 bp single-end reads, sequencing was performed on the Illumina iSeq 100 platform (USA) using a version 3 iSeq reagent kit. Quality control and microbiome data analysis was performed using the Quantitative Insights into Microbial Ecology (version 2021.08) pipeline. Amplicon sequence variants were identified and classified using the SILVA database (version 138). Microbial diversity was assessed using both alpha diversity metrics (Shannon and Inverse Simpson indices) and beta diversity measures (weighted UniFrac, unweighted UniFrac, and Bray-Curtis). Microbial composition was analyzed and visualized using R (version 4.3.2). A correlation analysis between the relative quantity of bacteria and tumor sizes was performed using GraphPad Prism 8 (GraphPad Software, USA).

### 2.15. Statistical analysis

All data are presented as means ± standard deviations (SDs) from a minimum of three independent experiments, each conducted in triplicate. Results were analyzed for statistically significant differences using Student's *t*-test (*), one-way, or two-way ANOVA (#). Statistical significance was defined as a *p*-value below 0.05 (*p < 0.05; **p < 0.01; ***p < 0.001; ^#^*p* < 0.05; ^##^*p* < 0.01; ^###^*p* < 0.001).

## 3. Results

### 3.1. Microbiome-derived metabolites spermine and sphingosine enhance sorafenib efficacy in HCC

**Figure 1:**
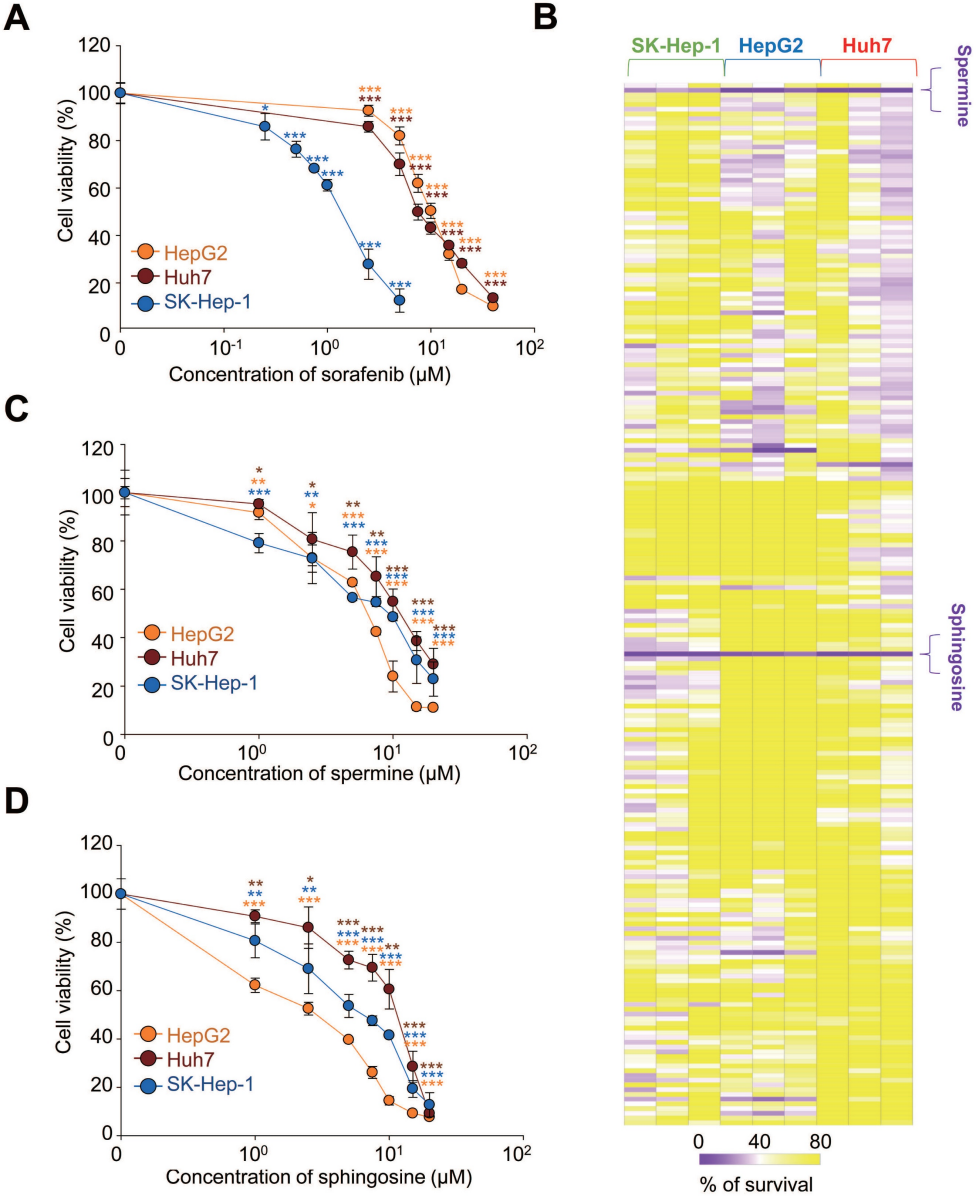
** Microbiome-derived metabolites spermine and sphingosine enhance sorafenib efficacy in hepatocellular carcinoma. (A)** Sorafenib was administered to HCC HepG2, Huh7, and SK-Hep-1 cells. The cell viability percentage was plotted after 48 hours of treatment. The GI_50_ value in each cell line was calculated based on the viability assay. **(B)** Cell viability was visualized as a heatmap after HepG2, Huh7, and SK-Hep-1 cells were treated with sorafenib (at the GI_50_ concentration for each cell line) and each of 220 microbiome-derived metabolites from a library at the concentration of 10 µM. The intensity was % of survival and ranged from 0 to 100. Spermine **(C)** and sphingosine **(D)** were administered to HepG2, Huh7, and SK-Hep-1 cells. The percentage of cell viability was plotted after 48 hours of treatment. The GI_20_, GI_30_, and GI_50_ values for each inhibitor in each cell line were calculated based on the viability assay. *p < 0.05; **p < 0.01; ***p < 0.001 compared with control. Data are presented as the mean ± SD.

Although sorafenib remains a cornerstone in the treatment of advanced HCC, its therapeutic benefits are limited, offering only modest survival improvements in advanced cases 13. To enhance sorafenib's efficacy, we explored microbial metabolites, which result from interactions between the host and microbiome, because they can exert local effects 23, 24. To account for the diverse characteristics of HCC, we tested three HCC cell lines: HepG2, Huh7, and SK-Hep-1. First, we determined the effective concentration range in which sorafenib has anticancer effects by using a cell viability assay across these cell lines with varying concentrations of sorafenib. The half maximal growth inhibitory concentration (GI_50_) values of sorafenib were 10.87 µM for HepG2, 7.65 µM for Huh7, and 1.33 µM for SK-Hep-1 (Figure 1A). Next, we investigated which of 220 different microbial metabolites (10 µM) effectively suppressed cell viability when sorafenib was co-administered at the GI_50_ concentration for each cell line (Figure 1B, Table 1). The results were visualized in a heatmap (Figure 1B). Among the tested metabolites, spermine and D-erythro-sphingosine (sphingosine) were the most effective and common metabolites across all three HCC cell lines, reducing cell viability to below 40% compared with sorafenib alone (Figure 1B, Table 1).

Because some level of cell viability was still observed with the combination treatment, we further analyzed the independent effects of spermine and sphingosine on each HCC HepG2, Huh7, and SK-Hep-1 cells. Both metabolites suppressed HCC cell growth in a concentration-dependent manner. The GI_50_ values of spermine were 8.2 µM for HepG2, 12.66 µM for Huh7, and 9.58 µM for SK-Hep-1 (Figure 1C). Similarly, the GI_50_ values of sphingosine were 2.17 µM for HepG2, 12.22 µM for Huh7, and 6.41 µM for SK-Hep-1 cells (Figure 1D). These results suggest that spermine and sphingosine not only enhance the efficacy of sorafenib but also possess intrinsic anti-HCC properties.

### 3.2. Combining sorafenib with spermine or sphingosine produces synergistic anti-cancer effects in advanced HCC

**Figure 2:**
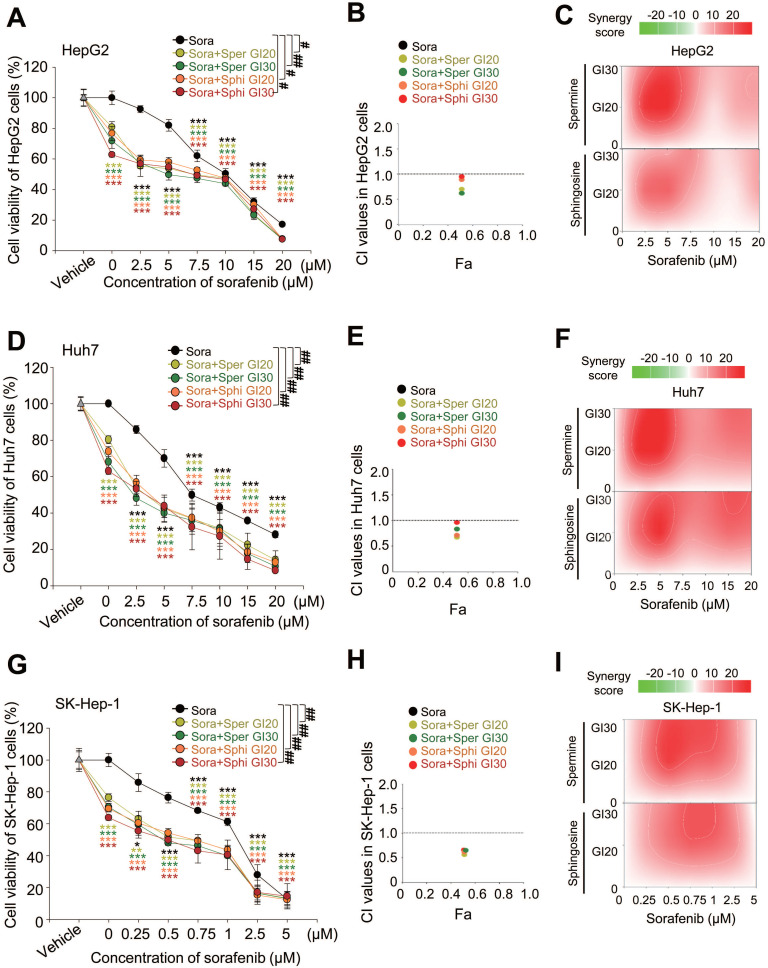
** Combining sorafenib with spermine or sphingosine produces synergistic anticancer effects in advanced hepatocellular carcinoma.** HepG2 **(A-C)**, Huh7 **(D-F)**, and SK-Hep-1 **(G-I)** cells were treated with the microbiome-derived metabolites spermine (Sper) or sphingosine (Sphi) at their GI_20_ or GI_30_ concentrations and different concentrations of sorafenib (Sora) for 48 hours. Using Compusyn software, the combination effects in HepG2 **(B)**, Huh7 **(E)**, and SK-Hep-1 **(F)** cells were calculated and displayed as the combination index (CI). Fa, Fraction affected. Using SynergyFinder software the combination effects in HepG2 **(C)**, Huh7 **(F)**, and SK-Hep-1 **(I)** cells were visualized. Student's *t*-test (*) or a two-way ANOVA (#) was performed to determine statistical significance. *p* < 0.05; **, *p* < 0.01; ***, *p* < 0.001 compared with control; #, *p* < 0.05; ##, *p* < 0.01 compared with the indicated group. Data are presented as the mean ± SD.

To evaluate how combining sorafenib with spermine or sphingosine affects HCC cells, we assessed cell viability following the combination treatments. Cells were treated with the GI_20_ or GI_30_ concentrations of spermine or sphingosine and sorafenib (Figure 2), and cell viability was assayed 48 h later. In HepG2 cells (derived from hepatoblastoma and representing well-differentiated HCC), spermine was administered at the GI_20_ (1.7 µM) and GI_30_ (2.5 µM) concentrations and varying concentrations of sorafenib. When sorafenib was combined with spermine at GI_20_, the GI_50_ of sorafenib was 6.3 µM, whereas with spermine at GI_30_, the GI_50_ of sorafenib decreased to 4.1 µM. Similarly, when sphingosine was co-administered at GI_20_ (0.47 µM) or GI_30_ (0.78 µM), the GI_50_ values of sorafenib were 7.69 µM and 6.45 µM, respectively (Figure 2A). To further evaluate the combined effects, the CI values were calculated. The CI values for sorafenib combined with spermine were approximately 0.79 (with a GI_20_ concentration of spermine) and 0.68 (with a GI_30_ concentration of spermine) in HepG2 cells (Figure 2A-B, Table 2). The CI values when sorafenib was combined with sphingosine were approximately 0.92 (with a GI_20_ concentration of sphingosine) and 0.95 (with a GI_30_ concentration of sphingosine) in HepG2 cells (Figure 2A-B, Table 2). When the combination effects were visualized using SynergyFinder, they displayed a similar pattern (Figure 2C). Because a CI value less than 1 indicates synergism and greater than 1 indicates antagonism, these results suggest that sorafenib combined with spermine exhibits a strong synergistic effect, whereas its combination with sphingosine shows a modest synergistic effect in HepG2 cells.

In Huh7 cells (moderately-differentiated HCC), spermine was administered at the GI_20_ (2.7 µM) and GI_30_ (6.2 µM) concentrations and varying concentrations of sorafenib. When sorafenib was combined with spermine at GI_20_, the GI_50_ of sorafenib was 3.34 µM, whereas at GI_30_, the GI_50_ of sorafenib was reduced to 2.5 µM. Similarly, when sphingosine at GI_20_ (6.2 µM) or GI_30_ (7 µM) was administered with sorafenib, the GI_50_ values were 3.65 µM and 3.04 µM, respectively (Figure 2D). To determine the combined effects, the final CI value was calculated. The CI values for sorafenib combined with spermine were approximately 0.65 (with a GI_20_ of spermine) and 0.81 (with a GI_30_ of spermine) in Huh7 cells (Figure 2D-E, Table 2). Likewise, the CI values when sorafenib was combined with sphingosine were approximately 0.69 (with a GI_20_ of sphingosine) and 0.97 (with a GI_30_ of sphingosine) in Huh7 cells (Figure 2D-E, Table 2). When the combination effects were visualized using SynergyFinder, synergistic effects were displayed with similar patterns (Figure 2F). Because a CI value of less than 1 indicates synergism, these results indicate that combining sorafenib with spermine or sphingosine at GI_20_ produces a strong synergistic effect in Huh7 cells.

In SK-Hep-1 cells (representing metastatic HCC), spermine was administered at the GI_20_ (1.08 µM) and GI_30_ (2.82 µM) concentrations and varying concentrations of sorafenib. When sorafenib was administered with spermine at GI_20_, the GI_50_ of sorafenib was 0.6 µM, whereas at GI_30_, it was reduced to 0.46 µM. Similarly, when sphingosine at GI_20_ (1.13 µM) or GI_30_ (2.02 µM) was administered with sorafenib, the GI_50_ values were 0.6 µM and 0.44 µM, respectively (Figure 2G, Table 2). To assess the combined effects, the final CI was calculated. The CI values for sorafenib combined with spermine were approximately 0.56 (at GI_20_) and 0.64 (at GI_30_) in SK-Hep-1 cells (Figure 2G-H, Table 2). Likewise, the CI values for sorafenib combined with sphingosine were approximately 0.63 (at GI_20_) and 0.65 (at GI_30_) in SK-Hep-1 cells (Figure 2G-H, Table 2). When visualized using SynergyFinder, the combination effects showed a similar pattern (Figure 2I), indicating that sorafenib combined with either spermine or sphingosine at any concentration exhibits strong synergism in SK-Hep-1 cells.

Therefore, cotreatment with the microbial metabolites spermine and sphingosine enhances the anti-tumorigenic effects of sorafenib in HCC, particularly in advanced cases, compared with sorafenib alone.

### 3.3. Combining sorafenib with spermine or sphingosine produces synergistic apoptotic effects in HCC through cell-cycle arrest

**Figure 3:**
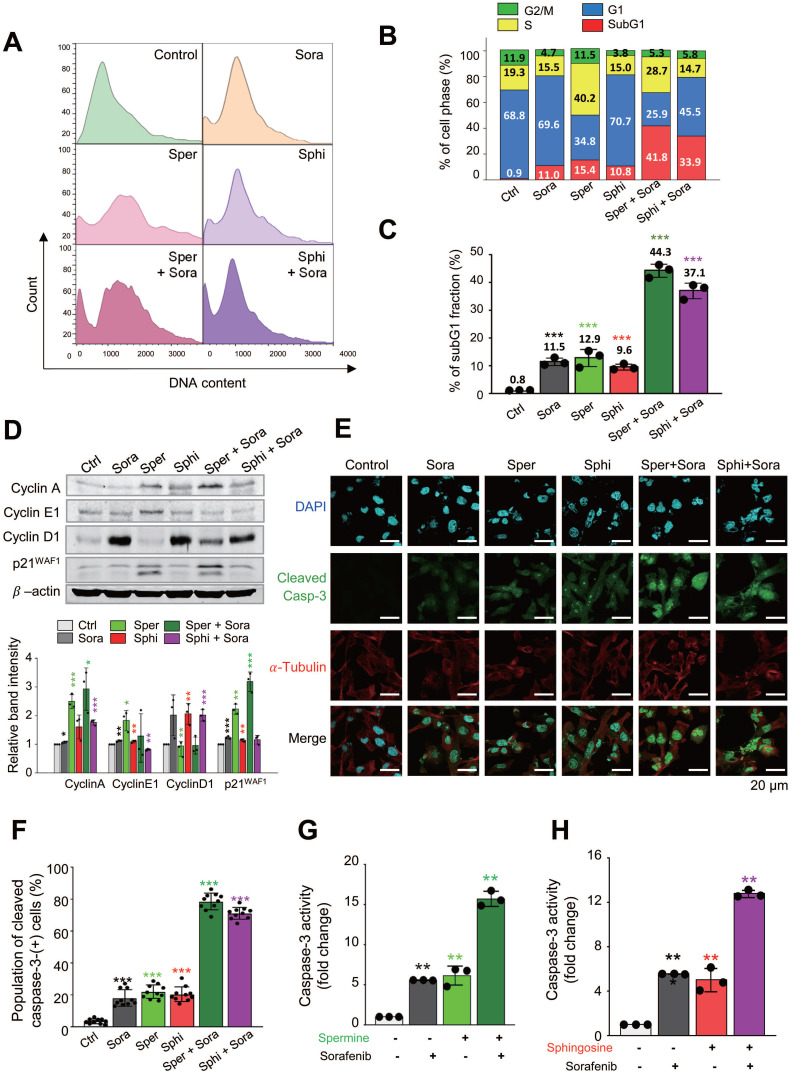
** Combining sorafenib with spermine or sphingosine produces synergistic apoptotic effects in hepatocellular carcinoma through cell-cycle arrest. (A)** A flow cytometry analysis was performed on SK-Hep-1 cells treated with sorafenib (Sora), spermine (Sper), and/or sphingosine (Sphi). **(B)** The percentage of cells in each cell-cycle phase was measured by flow cytometry, and the population of cells was plotted. SubG1, red; G1, blue; S, yellow; G2/M, green. **(C)** The percentage of cells in subG1 fraction was plotted. **(D)** Immunoblot analyses were performed on cells treated with sorafenib (Sora), spermine (Sper), and/or sphingosine (Sphi), either individually or in combination, using specific antibodies against cyclin A, cyclin E1, cyclin D1, p21^WAF1^, and β-actin. The relative band intensities were plotted. *p < 0.05; **p < 0.01; ***p < 0.001 compared with control.** (E)** Immunofluorescence staining was performed in cells after single or combination treatment with sorafenib (Sora), spermine (Sper), and/or sphingosine (Sphi). Cleaved caspase-3 (green), α-tubulin (red), and DNA (DAPI, blue) are displayed. n>3000. The cell images were collected and evaluated with a confocal microscope FW3000 (Olympus; Tokyo, Japan). Scale bar, 20 μm. (**F**) The population of cleaved caspase-3 (green fluorescence)-positive cells was quantified. n>3000. *p < 0.05; **p < 0.01; ***p < 0.001. Data are presented as the mean ± SD. (**G-H**) SK-Hep-1 cells were treated with sorafenib with and without spermine (**G**), or sorafenib with and without sphingosine (**H**) for 48 hours. Then the relative caspase-3 activity was measured with Ac-DEVD-AMC substrate and plotted. *p < 0.05; **p < 0.01; ***p < 0.001 compared with control. Data are presented as the mean ± SD.

To investigate how cell-cycle changes contribute to cell death following cotreatment with sorafenib and spermine or sphingosine, we used a flow cytometry analysis to analyze the cell-cycle distribution in SK-Hep-1 cells. Cells were treated with spermine, sphingosine, sorafenib, sorafenib plus spermine, or sorafenib plus sphingosine at their GI_30_ concentrations for 48 h (Figure 3A-C). Sorafenib treatment alone induced G1 arrest, with 69.6% of the cell population accumulating in the G1 phase. In contrast, treatment with spermine left 40.2% of the cells in the S phase. When spermine was combined with sorafenib, a significant increase in apoptotic cell death was observed, as indicated by a substantial rise in the subG1 fraction to 41.8% (Figure 3A-B). Similarly, treatment with sphingosine alone caused 70.7 % of cells to arrest in the G1 phase, which is similar to sorafenib. When sphingosine was combined with sorafenib, apoptotic cell death was significantly enhanced, with the subG1 fraction increasing to 33.9% (Figure 3A-B). Overall, the subG1 fraction increased dramatically, from approximately 10~15% with each single treatment to 41.8% with the sorafenib-spermine combination and 33.9% with the sorafenib-sphingosine combination in SK-Hep-1 cells (Figure 3B). The cell cycle patterns observed in Huh7 were similar in the FACS analysis (Figure S1A-C).

To further examine cell cycle arrest induced by sorafenib, spermine, or sphingosine, immunoblot analysis was performed in SK-Hep-1 cells. Treatment of sorafenib or sphingosine increased the expression of cyclin D1, a G1-phase cyclin, compared with the control (Figure 3D). In contrast, spermine treatment upregulated cyclin A (S-phase cyclin) and cyclin E1 (G1/S transition cyclin), consistent with the S-phase arrest observed in FACS analysis (Fig. 3A-B). The combination of sorafenib and spermine further increased cyclin A expression compared with single treatments. The CDK inhibitor p21^WAF1/CIP1^ was also induced during S-phase arrest by spermine (Figure 3D). Notably, spermine-induced S-phase arrest was accompanied by the accumulation of p21^WAF1/CIP1^ and the appearance of its cleavage fragment, suggesting a transition toward apoptosis.

Then to determine whether the observed cell death was apoptosis, immunostaining was performed with anti-cleaved caspase-3 antibody. The intensity of cleaved caspase-3 was significantly higher in cells cotreated with sorafenib and either spermine or sphingosine, compared with each single treatment. Specifically, the cleaved caspase-3 intensity increased up to 4.5-fold (single treatment of sorafenib vs. combination with spermine: 17 *vs.* 78) and 4.1-fold in combination (single treatment of sorafenib vs. combination with sphingosine: 17 *vs.* 70) compared with sorafenib alone (Figure 3E-F). In addition, compared with spermine or sphingosine administration alone, the cleaved caspase-3 intensity increased up to 3.7-fold (single treatment of spermine vs. combination: 21 *vs.* 78) and 3.5-fold (single treatment of sphingosine vs. combination: 20 *vs.* 70) in combination (Figure 3E-F). Thus, cotreatment with sorafenib and either spermine or sphingosine induces a much stronger, synergistic apoptotic response than any of the three treatments alone. The marked increase in cleaved caspase-3 further indicates the synergistic effects of these combinations in promoting cancer cell death.

To understand those findings in more detail, caspase-3 activity was analyzed using fluorogenic caspase-3 substrate in SK-Hep-1, Huh7, and HepG2 cells (Figure 3G-H, Figure S1D-G). The combination of sorafenib with either spermine or sphingosine led to a significant increase in caspase-3 activity, approximately 15.7- or 12.8-fold, respectively, higher than the control in SK-Hep-1 cells (Figure 3G-H). In cells cotreated with sorafenib and spermine, caspase-3 activity was approximately 2.8 times higher than with treatment with sorafenib (5.6 *vs.* 15.7) or spermine (6.1 *vs.* 15.7) alone (Figure 3G). Similarly, the combination of sorafenib and sphingosine resulted in an approximately 12.8-fold increase in caspase-3 activity compared with the control (Figure 3H). In this case, caspase-3 activity was approximately 2.3 times higher than with sorafenib alone (5.6 *vs.* 12.8) and 2.6 times higher than with sphingosine alone (5.0 *vs.* 12.8) (Figure 3H). These results further demonstrate that combining spermine or sphingosine with sorafenib produces a strong synergistic effect that significantly enhances apoptosis in SK-Hep-1 cells.

Similar patterns were observed in HepG2 and Huh7 cells (Figure S1D-G). In Huh7 cells, combining sorafenib with spermine or sphingosine produced approximately 13.5-fold or 11-fold increases in caspase-3 activity, respectively, compared with the control (Figure S1D-E). Notably, the combined treatment exhibited higher caspase-3 activity than the sum of the individual treatments (Figure S1D-E). A similar trend was observed in HepG2 cells, with cotreatment of sorafenib with spermine or sphingosine resulting in approximately 13-fold or 8-fold increases in caspase-3 activity, respectively, compared with the control (Figure S1F-G). Again, the combination treatment produced greater caspase 3 activity than the sum of the single treatments (Figure S1F-G). These results indicate that adding the microbial metabolites spermine or sphingosine to sorafenib treatment produces a stronger, synergistic effect on cancer cell death than the individual treatments. As shown by our FACS analysis of the subG1 fraction, immunostaining analysis, and caspase-3 activity assays, combining sorafenib with spermine or sphingosine produces potent synergistic effects in HCC cells.

### 3.4. Sorafenib treatment reduces the expression of catabolic enzymes (SMOX, CERS1, and SPHK1) involved in spermine and sphingosine metabolism via SP1 and HIF1a, whose depletion diminishes their synergistic effects

**Figure 4:**
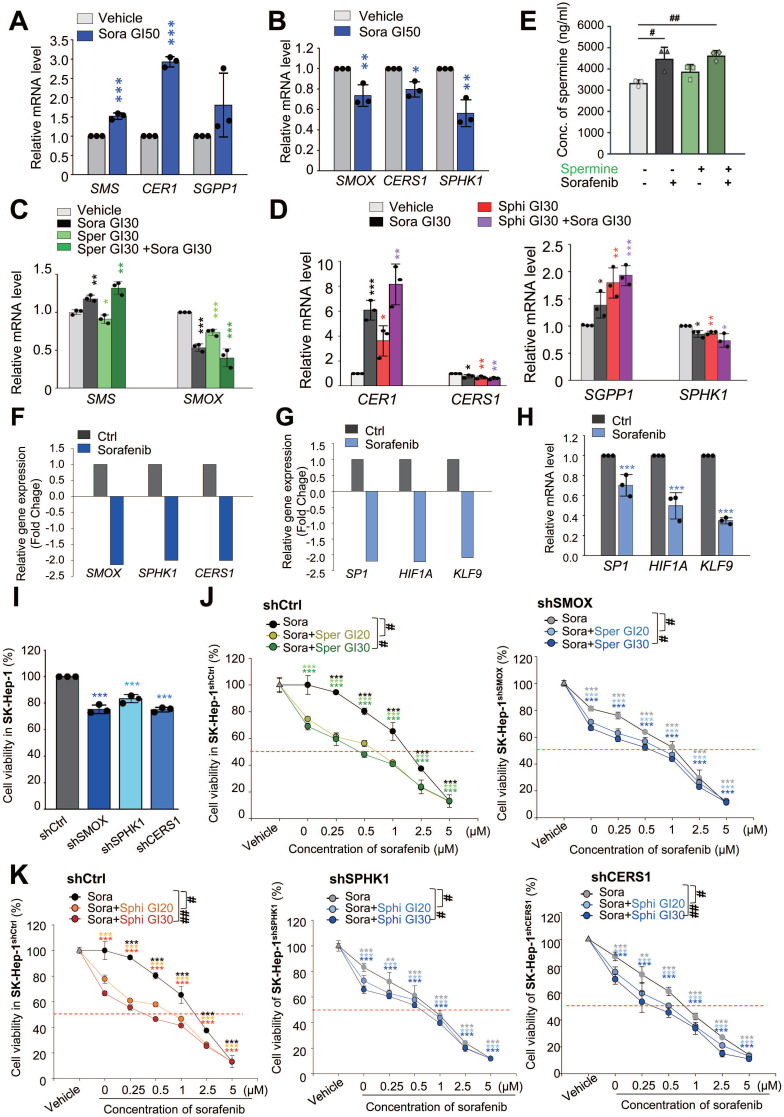
** Sorafenib treatment reduces the expression of catabolic enzymes (SMOX, SPHK1, and CERS1) involved in spermine and sphingosine metabolism via SP1 and HIF1a, whose depletion diminishes the synergistic effects. (A-B)** SK-Hep-1 cells were treated with sorafenib (Sora) for 48 hours. qRT-PCR analysis was performed to observe the expression of the synthetic enzymes *SMS*, *CER1*, and *SGPP1* **(A)** and the catabolic enzymes *SMOX*, *CERS1*, and *SPHK1* **(B)**. **(C-D)** SK-Hep-1 cells were treated with sorafenib (Sora) and spermine (Sper) (**C**) or sphingosine (Sphi) (**D**) for 48 hours. A qRT-PCR analysis was performed to observe the expression of the synthetic enzymes *SMS*, *CER1*, and *SGPP1* and the catabolic enzymes *SMOX*, *CERS1*, and *SPHK1*. **(E)** Intracellular spermine levels were measured by LC-MS analysis in SK-Hep-1 cells treated with sorafenib and/or spermine at the GI_30_ concentration for 48 hours. Data are presented as mean ± SEM (n = 3). Statistical significance was determined by one-way ANOVA. #, *p* < 0.05, ##, *p* < 0.01 versus control. **(F)** Using publicly available RNA-Seq data (GSE96794), the relative expressions of *SMOX*, *SPHK1*, and* CERS1* were analyzed and plotted.** (G)** Using publicly available RNA-Seq data (GSE96794), the relative expressions of predicted transcriptional factors *SP1*, *HIF1A*, and* KLF9* were analyzed and plotted. **(H)** A qRT-PCR analysis was performed using SK-Hep-1 cells of (**A**) panel to observe the expression of predicted transcriptional factors *SP1*, *HIF1A*, and* KLF9*. Their relative expressions were analyzed and plotted. ***p < 0.001 versus control.** (I)** Cell viability assay was assessed in SK-Hep-1 cells depleted of SMOX, SPHK1, or CERS1 using viral shRNA following puromycin selection for 48 hours. **(J)** Cell viability assay was performed in control (**left**) and SMOX-depleted (**right**) SK-Hep-1 cells treated with spermine or sphingosine at their GI_20_ or GI_30_ concentrations in combination with various concentration of sorafenib for 48 hours. **(K)** Cell viability was evaluated in control (**left**), SPHK1-depleted (**middle**), and CERS1-depleted (**right**) SK-Hep-1 cells treated with the spermine or sphingosine at their GI_20_ or GI_30_ concentrations in combination with various concentration of sorafenib for 48 hours. Student's *t*-test (*) or ANOVA (#) was performed to determine statistical significance. *, *p* < 0.05; **, *p* < 0.01; ***, *p* < 0.001 versus control. #, *p* < 0.05; ##, *p* < 0.01 compared with the indicated group. Data are presented as the mean ± SD.

Spermine and sphingosine are endogenous metabolites that play crucial roles in cellular functions 39, 40. Spermine is synthesized from spermidine by spermine synthase (encoded by *SMS*) and is metabolized by spermine oxidase (encoded by *SMOX*) (Figure S2A) 40. Sphingosine is derived from ceramide hydrolysis by ceramidase (encoded by *CER1*) and metabolized by ceramide synthase (encoded by *CERS1*). Additionally, sphingosine can be produced through the dephosphorylation of sphingosine-1-phosphate by sphingosine phosphatase (encoded by *SGPP1*) and metabolized by sphingosine kinase 1 (encoded by *SPHK1*) (Figure S2B) 39. To investigate the effects of sorafenib on the synthesis and metabolism of spermine and sphingosine, we measured the expression levels of their synthetic (*SMS, CER1,* and* SGPP1*) and metabolic (*SMOX, CERS1,* and* SPHK1*) enzymes at the half-maximal growth inhibitory concentration (GI_50_) of sorafenib using qRT-PCR (Figure 4A-B). In SK-Hep-1 cells, sorafenib treatment led to an upregulation of synthetic enzymes (*SMS, CER1,* and* SGPP1*) and a downregulation of metabolic enzymes (*SMOX, CERS1,* and* SPHK1*) (Figure 4A-B), indicating that sorafenib itself enhances endogenous levels of spermine and sphingosine by increasing synthetic enzymes and suppressing metabolic enzymes. In addition, co-administration of sorafenib and spermine or sphingosine at the concentration of GI_30_ increased the upregulation of the synthetic enzymes (*SMS, CER1,* and* SGPP1*) and downregulation of the metabolic enzymes (*SMOX, CERS1,* and* SPHK1*) compared to those of vehicle or sorafenib alone (Figure 4C-D). Notably, the combination of sorafenib with sphingosine synergistically enhanced the upregulation of the synthetic enzymes (*CER1* and* SGPP1*) and downregulation of the metabolic enzymes (*CERS1* and* SGPP1*) (Figure 4D). Thus, combining sorafenib with sphingosine or spermine suppressed their catabolic pathways and promoted their biosynthetic pathways, potentially leading to elevated endogenous levels of sphingosine or spermine.

Then to assess the intracellular levels of spermine or sphingosine, LC-MS/MS analysis was performed (Figure S2C, Figure 4E). As expected, intracellular spermine levels were elevated following sorafenib treatment (Figure 4E). In contrast, exogenous spermine treatment did not alter intracellular spermine levels, and cotreatment with sorafenib and spermine produced levels comparable to sorafenib alone. Intracellular sphingosine levels were too low to detect meaningful changes upon sorafenib treatment (Figure S2D).

We also examined how *SMOX*,* SPHK1*, and* CERS1* are regulated in sorafenib-treated HCC cells using the publicly available RNA-Seq dataset (GSE96794). As shown in Fig. 4F, the expressions of these genes were decreased (fold change < -1.5), consistent with expectations. Previous studies indicate that these genes are transcriptionally regulated by SP1 41-43, HIF1a 44, 45, and KLF9 46. Among them, SP1 is a plausible common regulator of three genes 41-43, while HIF1a may be shared for *SMOX* and* SPHK1* expression 44, 45. Consistent with the reports, RNA-Seq analysis showed that these transcription factors were also reduced (f.c. < -2.0; Fig. 4G). qRT-PCR showed the downregulation of SP1, HIF1a, and KLF9 to approximately 0.8-, 0.5-, and 0.3-fold, respectively (Fig. 4H). Thus, these data suggest that reduced levels of SP-1, HIF1a, and KLF9 may contribute to the downregulation of *SMOX*, *SPHK1*, and *CERS1* in sorafenib-treated HCC cells.

Next, to investigate the roles of SMOX, CERS1, and SPHK1 in synergism of sorafenib and the metabolites, the sorafenib-suppressed metabolic enzymes-*SMOX, CERS1,* and* SPHK1*-were silenced using shRNA (Figure S2E-J). Knockdown of these genes also reduced cell viability compared with control cells (Figure 4I, Figure S2E-J), indicating that their importance for the growth of HCC cells. Cotreatment with sorafenib and spermine or sphingosine at GI_20_ or GI_30_ concentration further decreased cell viability in *SMOX-*,* SPHK1-*, or *CERS1-*depleted SK-Hep-1 cells (Figure 4J-K, Table 3). Notably, *SMOX* depletion sensitized SK-Hep-1 cells to sorafenib or spermine treatment (Figure S2F). In SMOX-depleted cells, the GI_50_ value of sorafenib decreased from 1.44 µM (control cells) to 0.93 µM, and that of spermine decreased from 9.17 µM to 5.88 µM (Table S3). These results suggest that the synergistic effects of sorafenib and spermine are mediated, at least in part, through *SMOX* inhibition by sorafenib. Supporting this, the CI values for the sorafenib-spermine (GI_20_) combination increased from 0.54 to 0.91 µM in *SMOX*-depleted SK-Hep-1 cells (Figure 4J, Table 3), indicating that the synergism of sorafenib and spermine was diminished in the absence of SMOX. Similarly, in *SPHK1*-depleted cells, the GI_50_ values of sorafenib and sphingosine decreased from 1.44 µM (control cells) to 0.88 µM, and from 6.6 µM to 3.94 µM, respectively (Figure S2G-H, Table S3). The CI values of the sorafenib-sphingosine (GI_20_) combination increased from 0.71 to 0.94 (Figure 4K, Table 3), suggesting that their synergistic effects were reduced when *SPHK1* was silenced. Similar patterns were observed in *CERS1-*depleted cells (Figure S2I-J, Figure 4K, Table 3). Collectively, these findings indicate that the cooperative effect of sorafenib with spermine or sphingosine depend on *SMOX* or* SPHK1* and* CERS1*, respectively, highlighting their critical roles in HCC cell survival.

### 3.5. Clinical relevance of *SMOX*,* CERS1*, and *SPHK1* expression and combining effects of sorafenib with spermine or sphingosine in HCC organoids

**Figure 5:**
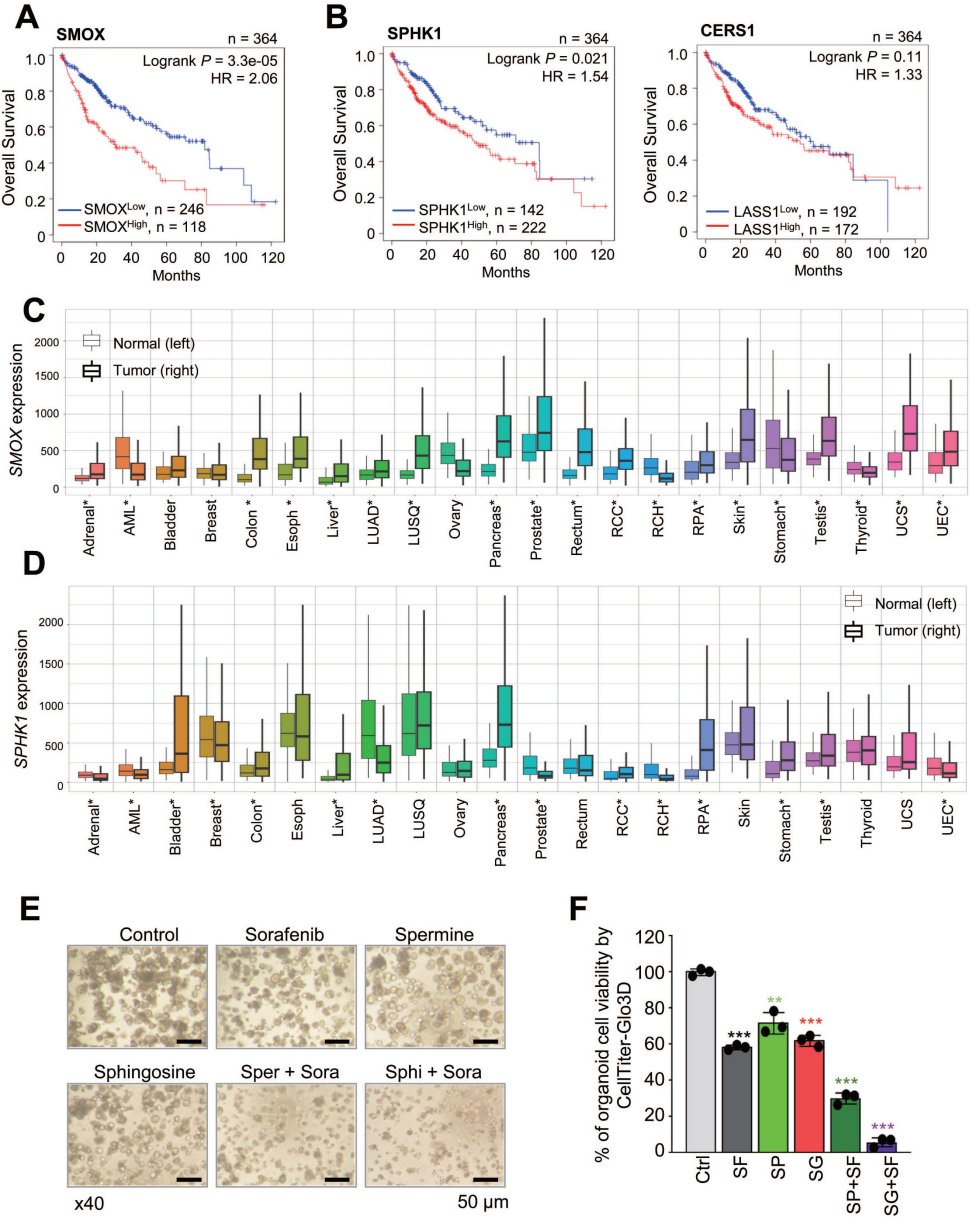
** Clinical relevance of *SMOX*,* CERS1*, and* SPHK1* expression and combining effects of sorafenib with spermine or sphingosine in HCC organoids. (A-B)** The overall survival rates of liver cancer patients in TCGA were plotted according to the expression levels of the *SMOX* metabolic enzyme of spermine **(A)** and the *CERS1* and *SPHK1* metabolic enzymes of sphingosine **(B)** using KM Plotter. **(C-D)** The relative gene expression of *SMOX* **(C)** and *SPHK1* **(D)** in normal (left) and tumor (right) tissues from adrenal cancer (Adrenal), acute myeloid leukemia (AML), bladder cancer (Bladder), breast cancer (Breast), colon cancer (Colon), esophageal cancer (Esoph), liver cancer (Liver), lung adenocarcinoma (LUAD), lung squamous cell carcinoma (LUSQ), ovary cancer (Ovary), pancreatic cancer (Pancreas), prostate cancer (Prostate), rectal cancer (Rectum), renal clear cell cancer (RCC), renal CH (RCH), renal PA (RPA), skin cancer (Skin), stomach cancer (Stomach), testis cancer (Testis), thyroid cancer (Thyroid), uterine CS cancer (UCS), and uterine EC (UEC). *: Mann-Whitney p<0.05 and expression >10 in tumor or normal tissue. **(E-F)** Patient-derived HCC SNU-423-CO organoids were treated with sorafenib with and without spermine or sphingosine for 6 days. **(E)** Representative images were obtained using a confocal microscope (TS100, Nikon, Tokyo, Japan). Scale bar, 50 μm. (**F**) Organoid formation efficiency and cell viability were assessed by CellTiter-Glo 3D assay. The relative organoid cell viability was plotted. Data are presented as the mean ± SD. **p < 0.01; ***p < 0.001.

To understand how the expression of these catabolic enzymes affects the survival rates of liver cancer patients, we analyzed overall survival (OS) with a Kaplan-Meier (KM) plotter using TCGA data (Figure 5A-B, Figure S3A-C, Table S4). Notably, patients with high expression levels of the spermine metabolic enzyme *SMOX* had significantly lower OS than those with low levels of *SMOX* (n = 364, Log rank *P* = 3.3e-05, HR = 2.06) (Figure 5A). Similarly, high expression of the sphingosine catabolic enzymes *SPHK1* and *CERS1* was associated with lower OS than low expression of *SPHK1* (n=364, Log rank *P* = 0.021, HR = 1.54) and* CERS1* (n = 364, Log rank *P* = 0.11, HR = 1.33) (Figure 5B). However, the expression patterns of the synthetic enzymes *SMS, CER1,* and* SGPP1* were inconsistent (Figure S3A-C). To further investigate the role of *SMOX, SPHK1,* and* CERS1* in various cancers, we analyzed their expression levels in cancerous vs. normal tissues (Figure 5C-D, Figure S3D). Among them, *SMOX* and* SPHK1* were highly expressed in multiple malignancies: acute myeloid leukemia (AML), liver, stomach, colon, lung, pancreas, prostate, testis, kidney, and other cancers (Figure 5C-D). These findings suggest that sorafenib treatment downregulates the expression of catabolic enzymes involved in spermine and sphingosine catabolism, *SMOX, SPHK1* and *CERS1,* whose expression is inversely correlated with the survival rates of liver cancer patients.

To evaluate the clinical potential of combining spermine or sphingosine with sorafenib in HCC, patient-derived HCC SNU-423-CO organoids were treated with each compound. GI_30_ concentration was determined by exposing HCC organoids to increasing doses of sorafenib, spermine, or sphingosine (Figure S4A-C), and organoids cell viability was measured using cell counting and CellTiter-Glo3D assay. Using these determined GI_30_ concentrations, combination treatments led to more than a twofold reduction in both organoid number and ATP activity compared with single treatments (Figure 5E-F, Figure S4D-E). These results demonstrate that combining sorafenib with spermine or sphingosine enhances anti-HCC efficacy in patient-derived HCC organoids.

### 3.6. Combining sorafenib with spermine or sphingosine produces synergistic anti-tumorigenic effects in xenograft mouse model of hepatocellular carcinoma

**Figure 6:**
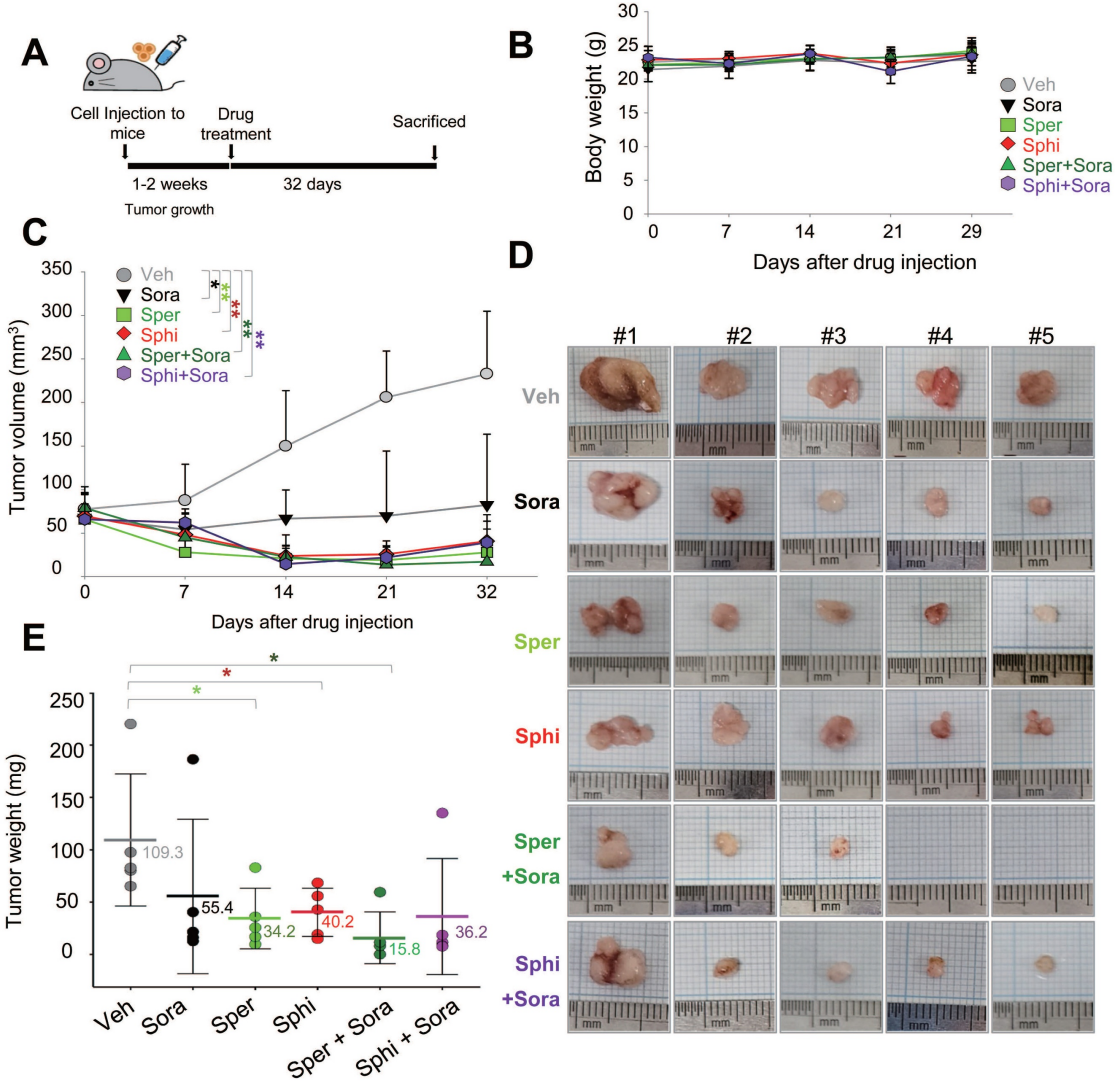
** Combining sorafenib with spermine or sphingosine produces synergistic anti-tumorigenic effects in xenograft mouse model of hepatocellular carcinoma. (A)** Scheme of the *in vivo* experiment. SK-Hep-1 cells were mixed with Matrigel and injected into the upper left thighs of mice. After one week, when tumor volumes reached 60 to 80 mm³, the 30 mice were randomly divided into six groups. Each group received one of the following treatments orally for 32 days: vehicle, sorafenib, spermine, sphingosine, sorafenib plus spermine, or sorafenib plus sphingosine. **(B)** The body weights of the mice were measured every week and plotted. **(C)** The tumor volume was measured for 32 days after treatment. **(D)** A representative xenograft tumor from the mouse model (n=5 per group). **(E)** Tumor weights of the mice were plotted. *p < 0.05; **p < 0.01 versus control.

To evaluate the *in vivo* efficacy of combining spermine or sphingosine with sorafenib, SK-Hep-1 cells were mixed with Matrigel and injected into the upper left thighs of mice. One week after tumor implantation, when tumor volumes reached 60 to 80 mm³, 30 mice were randomly divided into six groups and assigned to receive vehicle, sorafenib, spermine, sphingosine, sorafenib plus spermine, or sorafenib plus sphingosine (Figure 6A). The results demonstrate a remarkable reduction in tumor volume in mice treated with the combinations, with no significant changes in body weight (Figure 6B). The strongest tumor suppression was observed in the group receiving sorafenib and spermine (Figure 6C-E). Notably, tumors became undetectable in two mice approximately 14 days after they started the combination therapy (Figure 6C-E). Upon laparotomy, complete tumor disappearance was confirmed in these two mice. In the remaining mice in that combination group, tumor suppression was significantly greater than in mice treated with either sorafenib or spermine alone (Figure 6E). The average tumor weights were 109.3 mg (vehicle), 55.4 mg (sorafenib), 34.2 mg (spermine), and 15.8 mg (sorafenib plus spermine). Similarly, the combination of sorafenib and sphingosine reduced the tumor sizes to approximately 36.2 mg, which was much lower than with either sorafenib (55.4 mg) or sphingosine alone (40.2 mg) (Figure 6E). Therefore, combining sorafenib with spermine or sphingosine exhibited the greater efficacy in suppressing HCC growth in a xenograft mouse model.

### 3.7. Combining sorafenib with spermine or sphingosine alters the gut microbiome, increasing the relative abundance of *Faecalibaculum,* which is inversely correlated with tumor sizes in a xenograft mouse model of HCC

**Figure 7:**
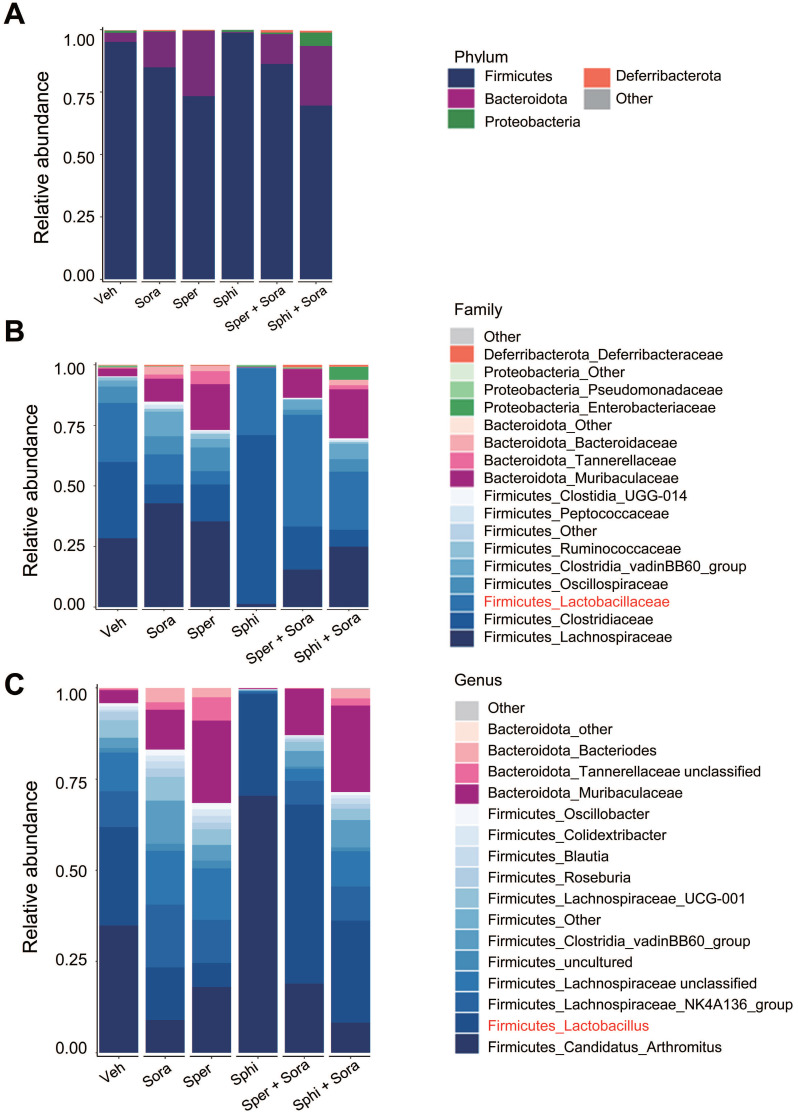
** Combining sorafenib with spermine or sphingosine alters the gut microbiome in a xenograft mouse model of hepatocellular carcinoma.** Cecal samples were collected from mouse intestines, and DNA was extracted. The bacterial 16S rRNA gene (V4 region) was amplified, and the microbiome sequencing data were analyzed. Relative abundance of gut microbiota **(A)** at the phylum level, **(B)** at the family level, and **(C)** at the genus level in those cecal samples (n = 5 per group).

**Figure 8:**
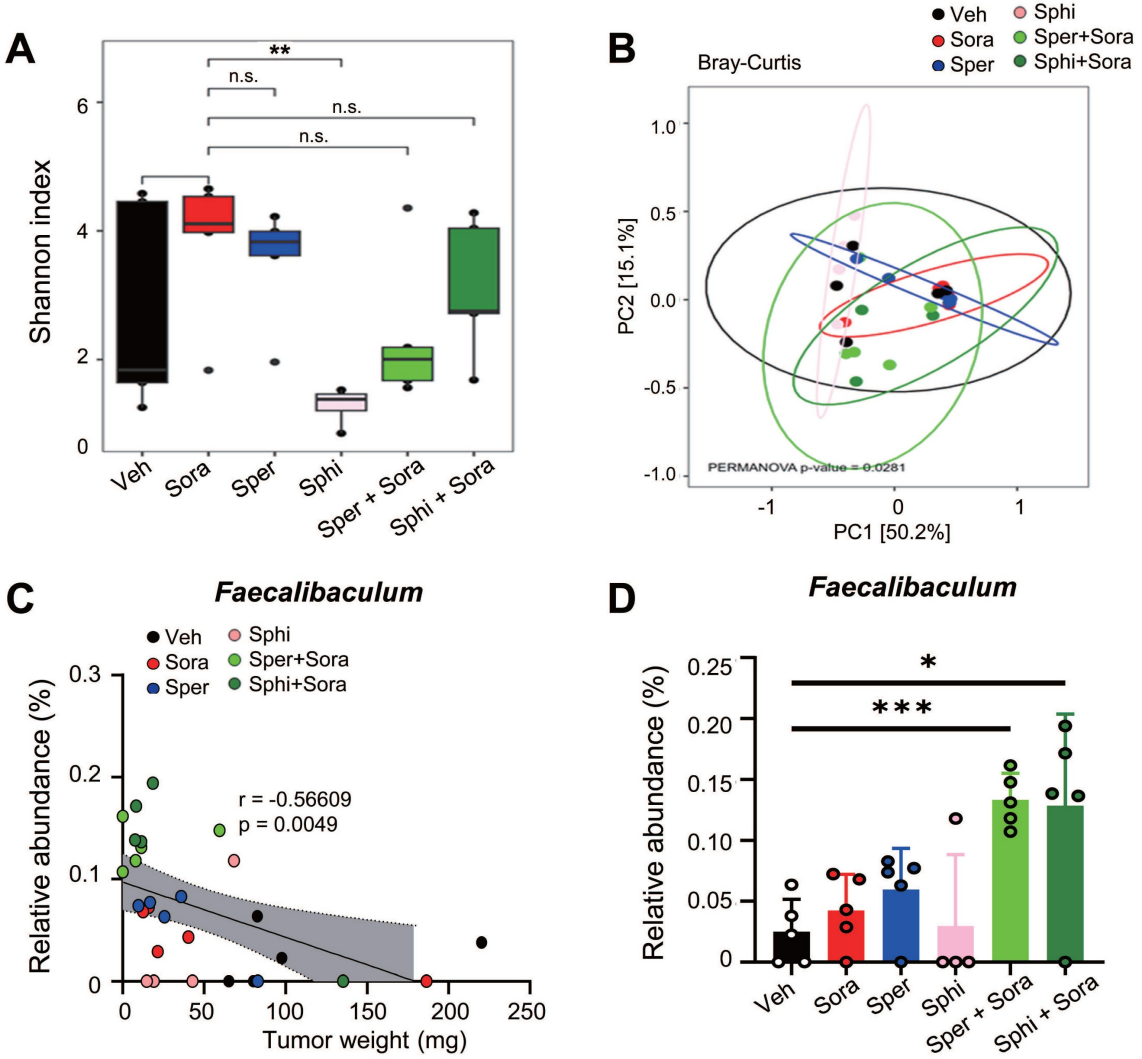
** Combining sorafenib with spermine or sphingosine increases the relative abundance of *Faecalibaculum*, a bacterial genus inversely correlated with tumor sizes in the xenograft mouse model of hepatocellular carcinoma.** Alpha diversity (Shannon and Inverse Simpson indices), beta diversity (weighted UniFrac, unweighted UniFrac, and Bray-Curtis), and microbial composition plots were analyzed. **(A)** Alpha diversity (α-diversity) as analyzed using the Shannon index (n = 5 per group). **(B)** Beta diversity (β-diversity) as analyzed using Bray-Curtis dissimilarity. **(C)** The correlation between the relative abundance of *Faecalibaculum* and tumor size was analyzed and plotted. **(D)** The relative abundance of *Faecalibaculum* was analyzed and plotted. *p < 0.05; ***p < 0.001.

To investigate the effects of sorafenib, spermine, and sphingosine treatments on gut microbiome composition, we performed 16S rRNA sequencing and analyzed microbiome diversity at the phylum, family, and genus levels (Figure 7A-C). The combination treatment with sorafenib and spermine or sphingosine increased the proportion of *Lactobacillaceae* at the family level (Figure 7B, Figure S5A) and *Lactobacillus* at the genus level (Figure 7C, Figure S5B). Microbial diversity was assessed using alpha and beta diversity indices. Alpha diversity, as assessed by the Shannon index (Figure 8A) and InvSimpson index (Figure S6A), tended to be higher in the vehicle group compared to the treated groups, although the differences were not statistically significant. Beta diversity analysis based on Bray-Curtis (Figure 8B), weighted UniFrac (Figure S6B), and unweighted UniFrac (Figure S6C) distances suggested a tendency for group-wise separation. However, PERMANOVA did not indicate statistically significant differences among the groups. Notably, a further correlation analysis between gut microbiome composition and tumor size in the xenograft mice revealed an inverse relationship between the relative abundance of *Faecalibaculum* and tumor size (Figure 8C-D). These data suggest that increased levels of *Faecalibaculum* are associated with tumor suppression. Thus, this microbial marker could have potential prognostic significance for HCC.

## 4. Discussion

**Figure 9:**
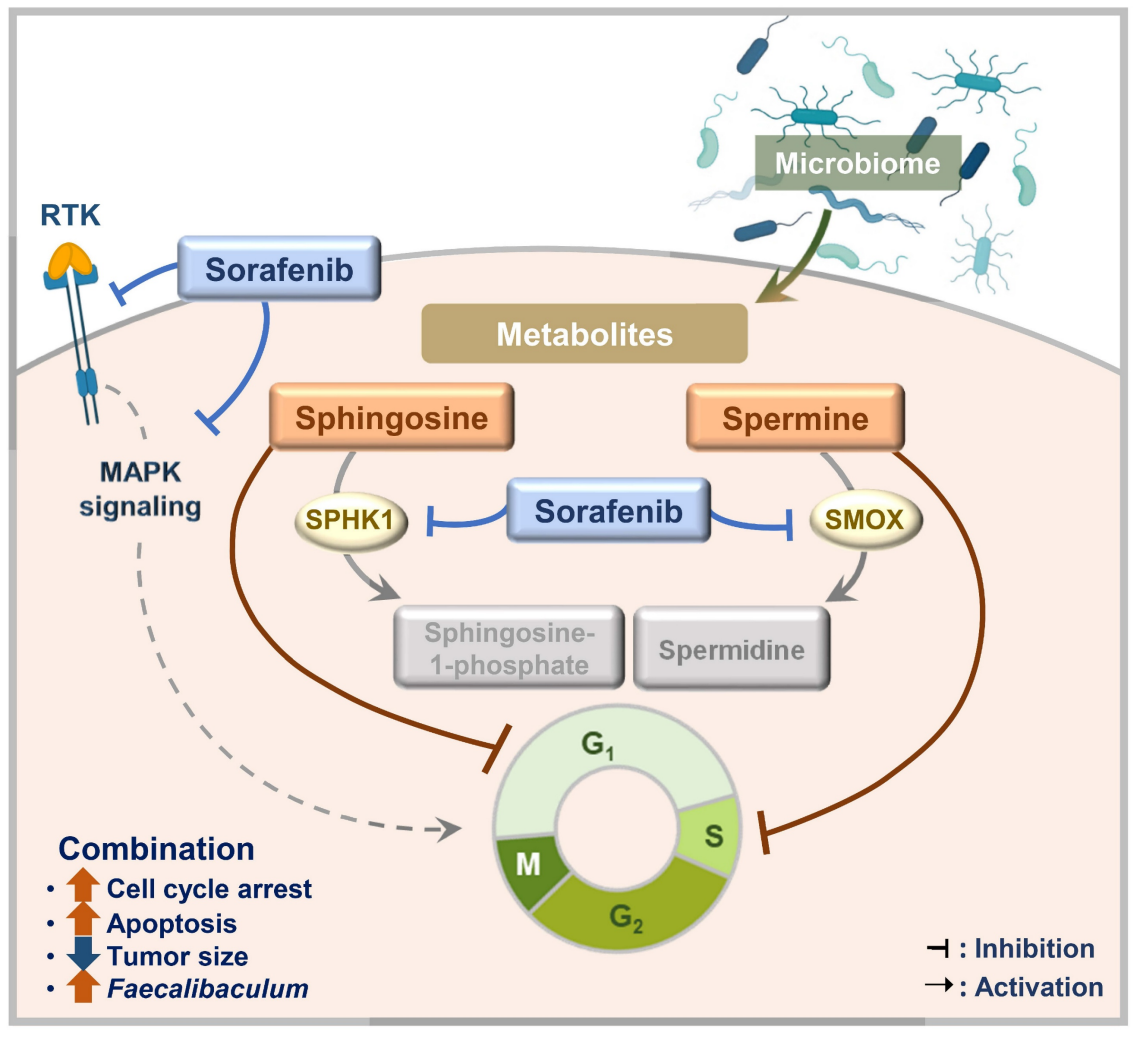
** A schematic model illustrating the enhanced sorafenib's efficacy through co-administration with spermine and sphingosine in HCC.** Combining sorafenib with microbiome-derived metabolite spermine or sphingosine enhanced its anti-HCC activity by inducing cell cycle arrest at G1 or S phase, ultimately leading to increased apoptosis. In parallel, sorafenib suppressed the expression of *SMOX* (a key catabolic enzyme for spermine), as well as *SPHK1* and *CERS1* (critical enzymes involved in sphingosine metabolism), whose elevated levels are linked to poor survival outcomes in liver cancer patients. In a xenograft model, the combination therapy also showed a clear inverse correlation between tumor size and the abundance of *Faecalibaculum*, pointing to its possible role as a prognostic gut microbiome marker for HCC.

A complex and multidisciplinary approach is needed to treat HCC because it is driven by various primary carcinogens. Sorafenib remains a basis of HCC treatment, but it has limitations, including a relatively modest survival benefit in advanced HCC 13. To address the limitations of sorafenib, we tested microbial metabolites, which result from interactions between the host and gut microbiome, to see whether they had synergistic anticancer effects with sorafenib on HCC. In this study, we found that spermine and sphingosine are sorafenib-efficacy-enhancing microbiome-derived metabolites with anti-HCC effects of their own. Their synergistic effect with sorafenib can be explained by the fact that spermine and sphingosine induce cell-cycle arrest at S and G1 phases, respectively. Consequently, combining sorafenib with spermine or sphingosine synergistically enhances apoptosis. In addition, we found that sorafenib regulates the metabolic and synthetic enzymes of spermine and sphingosine. Specifically, sorafenib treatment led to the downregulation of *SMOX* (a key catabolic enzyme for spermine), as well as *SPHK1* and* CERS1* (genes involved in sphingosine metabolism), whose high expression levels are associated with poorer survival outcomes in liver cancer patients according to TCGA data analysis. Furthermore, a 16S rRNA sequencing analysis revealed that combination of sorafenib with spermine or sphingosine alters the gut microbiome, increasing the relative abundance of *Faecalibaculum,* inversely correlated with tumor sizes in a xenograft mouse model. These findings suggest that *Faecalibaculum* may serve as a potential microbiome-based prognostic marker for predicting HCC progression, as its abundance is inversely correlated with tumor sizes. Therefore, we propose that combining sorafenib with microbiome-derived metabolites spermine or sphingosine synergistically enhances its anti-HCC effects by promoting cell-cycle arrest, suppressing the expression of key metabolic enzymes, and modulating gut microbiome composition in HCC (Figure 9).

Many previous studies reported that microbiome-derived metabolites affect cancer progression and drug responsiveness 47, but no previous research reported that spermine and sphingosine show synergistic effects with sorafenib. In our screen of a microbiome metabolite library, we found spermine and sphingosine as common metabolites that enhanced sorafenib efficacy in HCC cells with different characteristics. The genetic backgrounds of the HCC cell lines used in this study may contribute to their distinct therapeutic responses. HepG2 exhibits both genetic features consistent with hepatoblastoma and HCC, carrying a TERT promoter (C228T) mutation and wild-type TP53 that is generally associated with lower malignancy 48. In contrast, Huh7 cells harbor a TP53 mutation, commonly linked to higher malignancy 49. SK-Hep-1 cells possess mutations in both BRAF oncogene, a main component of the MAPK signaling pathway, and CDKN2A tumor suppressor gene, alterations that together promote metastatic potential 50. Different HCC cell lines exhibit varying levels of sensitivity to sorafenib and the selected microbial metabolites. The CI of sorafenib with spermine or sphingosine at GI_20_ was lowest in SK-Hep-1 cells (metastatic cells), medium in Huh7 cells (moderately differentiated, grade 2), and highest in HepG2 cells (well differentiated, grade 1), indicating that the combination of sorafenib with spermine or sphingosine would be most effective in advanced HCC.

This study is significant in that it suggests a metabolic control strategy with the potential to address the limitations of sorafenib monotherapy. Sorafenib treatment upregulated the expression of synthetic enzymes for spermine and sphingosine (*SMS*, *CER1*, and *SGPP1*). Spermine synthase (encoded by *SMS*) synthesizes spermine from spermidine 40. Ceramidase (encoded by *CER1*) produces sphingosine from ceramide, and sphingosine-1 phosphate phosphatase (encoded by *SGPP1*) drives the reaction from sphingosine-1 phosphate to sphingosine At the same time, sorafenib treatment suppressed the expression of metabolic enzymes of spermine and sphingosine (*SMOX*, *CERS1*, and *SPHK1*). Spermine oxidase (encoded by *SMOX*) degrades spermine to spermidine 40. Ceramide synthase (encoded by *CERS1, GDF1*) degrades sphingosine to ceramide, and sphingosine-1 phosphate kinase (encoded by *SPHK1*) phosphorylates sphingosine to produce sphingosine-1 phosphate 39. Clinically, the overall survival rates of liver cancer patients were inversely correlated with the expression levels of these metabolic enzymes: *SMOX* (n=364, Log rank P =3.3e-05, HR=2.06),* SPHK1* (n=364, Log rank P =0.021, HR=1.54), and *CERS1* (n=364, Log rank P =0.11, HR=1.33). Although the correlation between the overall survival rates of liver cancer patients and the expression of* CERS1* (n=364, Log rank P =0.11, HR=1.33) is not statistically significant, the *SMOX* and* SPHK1* correlations are significant. High *SMOX* and* SPHK1* expression is not limited to liver cancer, being found in AML and other carcinomas, including those of the colon, stomach, pancreas, lung, prostate, and testis. Therefore, sorafenib's suppressive effects on *SMOX* and* SPHK1* could be adapted to other carcinomas.

The results of this study suggest that combining sorafenib treatment with spermine or sphingosine could potentially improve the therapeutic responses in HCC patients. However, several major clinical challenges remain, including determining safety, optimal dosage, pharmacokinetics, and tissue distribution. Furthermore, potential microbiome alterations have to be considered, because both spermine and sphingosine are microbiome-derived metabolites. Existing pharmacokinetic data 51, 52 show that radioactive spermine accumulated at high levels in the kidney, likely due to renal excretion in rats 51, and had a half-life of approximately 24 hours in mouse fibroblasts 52. Sphingosine tracer studies showed that distribution to the skin and a Tmax of 10.7 hours in mouse blood 53. Sphingosine was also detected in the liver, kidney, spleen, and lung in murine tissues 54. In this study, we additionally found that combining sorafenib with spermine or sphingosine increased the relative abundance of *Faecalibaculum*, bacteria inversely correlated with tumor sizes in our xenograft mouse model of HCC. *Faecalibaculum* is a genus of gut bacteria that has been studied for its potential role for cancer therapy due to its anti-inflammatory properties 55, enhancement of the tumor-suppressive effects of dual CTLA4 and PD-1 immune checkpoint inhibitors 22, and its inhibition of tumor cell proliferation through the production of short-chain fatty acids 56. Therefore, the anti-HCC effects of combining sorafenib with spermine or sphingosine might be due to the modulation of the gut microbiome composition and the increase in *Faecalibaculum*. Further studies are required to determine whether it directly or indirectly regulates spermine or sphingosine metabolism and thereby influences therapeutic response. Additional analyses of tumor-infiltrating immune cells (e.g., Tregs and CD8⁺ T cells) will also be valuable to elucidate how immune responses interact with microbiota alterations in HCC. Despite promising preclinical results, the pharmacokinetic limitations of spermine and sphingosine may hinger their therapeutic use. To address these challenges, nanoparticle-based delivery strategies could be considered to enhance stability and safety. Advanced drug delivery strategies could be leveraged to overcome these limitations of spermine and sphingosine. Moreover, because *Faecalibaculum* abundance varies among individuals and may be shaped by diet or antibiotic use, large-scale studies are needed to establish its value as a robust biomarker in HCC. Overall, combining sorafenib with microbiome-derived metabolite spermine or sphingosine enhanced its anti-HCC activity by inducing cell cycle arrest at G1 or S phase, ultimately leading to increased apoptosis. In parallel, sorafenib suppressed the expression of spermine oxidase (a key catabolic enzyme for spermine), as well as sphingosine kinase 1 and ceramide synthase 1 (critical enzymes involved in sphingosine metabolism), whose elevated levels are linked to poor survival outcomes in liver cancer patients. In a xenograft model, the combination therapy also showed a clear inverse correlation between tumor size and the abundance of *Faecalibaculum*, pointing to its possible role as a prognostic gut microbiome marker for HCC.

## Supplementary Material

Supplementary figures and tables.

## Figures and Tables

**Table 1:** GI_20_, GI_30_, and GI_50_ values of sorafenib, spermine, and sphingosine in HCC cells.

Compound	Sorafenib (μM)			Spermine (μM)			Sphingosine (μM)		
	GI_20_	GI_30_	GI_50_	GI_20_	GI_30_	GI_50_	GI_20_	GI_30_	GI_50_
**Huh7**	2.9	4.23	**7.65**	2.7	6.2	**12.66**	2.6	7	**12.22**
**HepG2**	3.93	5.21	**10.87**	1.7	2.5	**8.2**	0.47	0.78	**2.17**
**SK-Hep-1**	0.48	0.67	**1.33**	1.08	2.82	**9.58**	1.13	2.02	**6.41**

**Table 2:** The half maximal growth inhibitory concentration (GI_50_) values of sorafenib, spermine, and sphingosine and the combination index (CI) values in hepatocellular carcinoma (HCC) cells. CI < 1 represents synergism, CI = 1 represents additive effect, and CI > 1 represents antagonism.

Compound	Huh7		HepG2		SK-Hep-1	
	GI_50_ (μM)	CI	GI_50_ (μM)	CI	GI_50_ (μM)	CI
**Sorafenib** (μM)	7.65		10.87		1.33	
**Spermine** (μM)	12.66		8.2		9.58	
**Sphingosine** (μM)	12.22		2.17		6.41	
**Sorafenib** in combination GI_20_ **Spermine**	3.34	0.65	6.3	0.79	0.6	0.56
**Sorafenib** in combination GI_30_ **Spermine**	2.5	0.81	4.1	0.68	0.46	0.64
**Sorafenib** in combination GI_20_ **Sphingosine**	3.65	0.69	7.69	0.92	0.6	0.63
**Sorafenib** in combination GI_30_ **Sphingosine**	3.04	0.97	6.45	0.95	0.44	0.65

**Table 3:** The half maximal growth inhibitory concentration (GI_50_) values of sorafenib, spermine, and sphingosine and the combination index (CI) values in SK-Hep-1^shCtrl^, SK-Hep-1^shSMOX^, SK-Hep-1^shSPHK1^ and SK-Hep-1^shCERS1^ cells. CI < 1 represents synergism, CI = 1 represents additive effect, and CI > 1 represents antagonism.

Compound	SK-Hep-1^shCtrl^		SK-Hep-1^shSMOX^		SK-Hep-1^shSPHK1^		SK-Hep-1^shCERS1^	
	GI_50_ (μM)	CI	GI_50_ (μM)	CI	GI_50_ (μM)	CI	GI_50_ (μM)	CI
**Sorafenib** (μM)	1.44		0.93		0.88		0.63	
**Spermine** (μM)	9.17		5.88					
**Sphingosine** (μM)	6.6				3.94		4.26	
**Sorafenib** in combination GI_20_ **Spermine**	0.6	0.54	0.75	0.91				
**Sorafenib** in combination GI_30_ **Spermine**	0.47	0.60	0.61	0.87				
**Sorafenib** in combination GI_20_ **Sphingosine**	0.64	0.71			0.61	0.94	0.46	0.98
**Sorafenib** in combination GI_30_ **Sphingosine**	0.41	0.69			0.51	0.98	0.33	0.92

## Abbreviations

DMEM: Dulbecco's modified Eagle's medium
FACS: Fluorescence-activated cell sorting
FBS: Fetal bovine serum
GI_50_: The half maximal growth inhibitory concentration
HCC: Hepatocellular carcinoma
HR: Hazard ratio
qRT-PCR: Quantitative reverse transcription polymerase chain reaction
Sphingosine: D-erythro-sphingosine
