# Supplementary material for: Combining sorafenib with spermine and sphingosine synergistically enhances anticancer efficacy by modulating metabolic pathways and gut microbiome in hepatocellular carcinoma
Source: Int J Biol Sci. 2026 Jan 1;22(3):1082–102. doi: 10.7150/ijbs.118753 (PMC12836515; doi:10.7150/ijbs.118753)
Supplement: Supplementary file 1 — Supplementary figures and tables. [file ijbsv22p1082s1.pdf]

Combining sorafenib with spermine and sphingosine synergistically enhances anticancer efficacy by modulating metabolic pathways and gut microbiome in hepatocellular carcinoma

Hay-Ran Jang[1,†], Hyun-Jin Kim[1,†], Bo-Young Kim[1], Jae-Hoon Jeong[2], Jeon-Kyung Kim[2], Jin Ah Won[1,3], Hye Hyun Yoo[1,3], Yong Gu Lee[1,3], and Hyungshin Yim[1,3,*]

[1]Department of Pharmacy, College of Pharmacy, Hanyang University, Ansan, Republic of Korea

[2]School of Pharmacy, Institute of New Drug Development, Jeonbuk National University, Jeonju, Republic of Korea

[3]Pharmacomicrobiomics Research Center, College of Pharmacy, Hanyang University, Ansan, Republic of Korea

[*]Corresponding author
Phone: +82-31-400-5810
Fax: +82-31-400-5958
E-mail: hsyim@hanyang.ac.kr

[†]The authors contributed equally.

Supplementary Figure S1

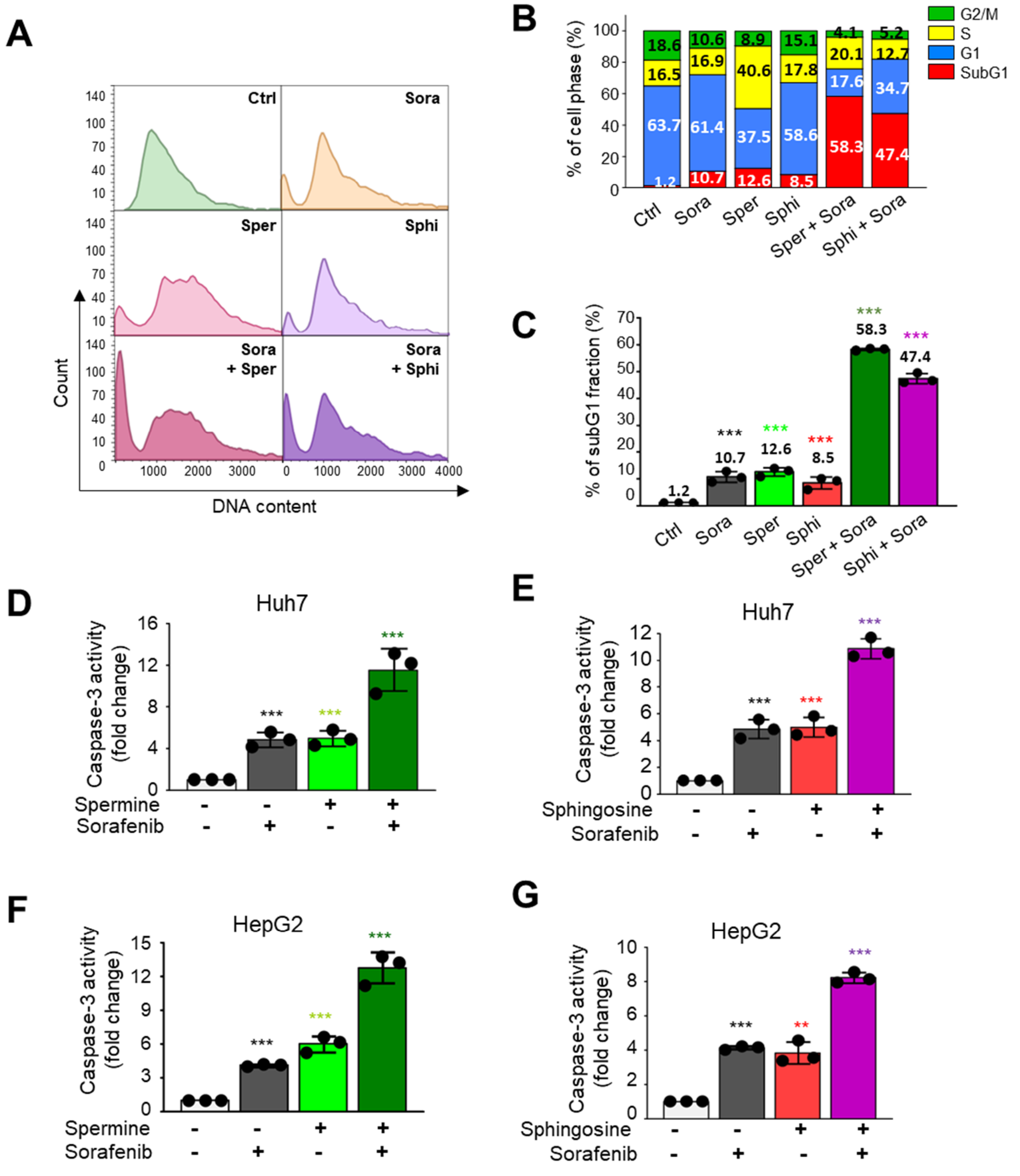

Figure S1. Combining sorafenib with spermine or sphingosine produces synergistic apoptotic effects in Huh7 and HepG2 cells through cell-cycle arrest. (**A–E**) Huh7 cells were treated with sorafenib and/or spermine or sphingosine for 48 hours. (**A–C**) A flow cytometry

analysis was performed on Huh7 cells treated with sorafenib (Sora), spermine (Sper), and/or sphingosine (Sphi). **(B)** The percentage of cells in each cell-cycle phase was measured, and the populations of cells were plotted. SubG1, red; G1, blue; S, yellow; G2/M, green. **(C)** The subG1 fraction cells was plotted. **(D-E)** The relative caspase-3 activity in Huh7 cells was measured with Ac-DEVD-AMC substrate and plotted. ***$p < 0.001$. Data are presented as the mean ± SD. (**F–G**) HepG2 cells were treated with sorafenib and/or spermine or sphingosine for 48 hours. The relative caspase-3 activity in HepG2 cells was measured with Ac-DEVD-AMC substrate and plotted. **$p < 0.01$, ***$p < 0.001$. Data are presented as the mean ± SD.

Supplementary Figure S2

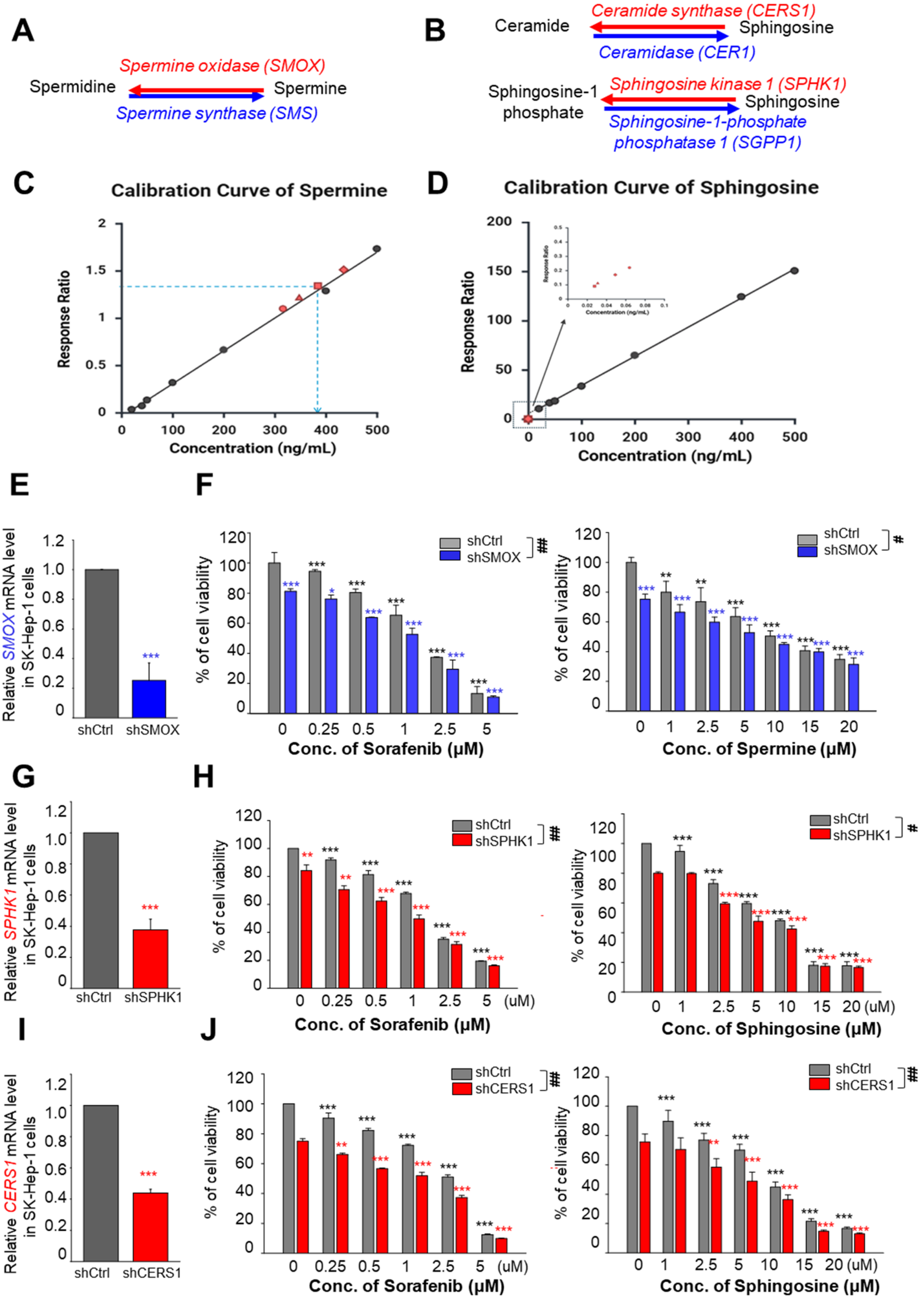

Figure S2. The survival rates of liver cancer patients according to the expression of the spermine synthase, ceramidase, and sphingosine-1-phosphate phosphatase 1 synthetic enzymes. (A-B) Main synthetic and metabolic pathways for spermine **(A)** and sphingosine **(B). (C-D)** For LC-MS analysis, standard calibration curves for spermine (**C**) and sphingosine (**D**) were plotted for quantification showing the linear relationship between concentration (ng/ml) and response ratio. Response ratio was calculated from dividing analyte peak area by internal standard (terfenadine) peak area. Calibration ranges for spermine and sphingosine were 20–500 ng/ml and 10–500 ng/ml, respectively. The blue dashed lines and arrow in (**C**) indicate the representative sample concentration determination. Inset in (**D**) shows the low concentration range (0.025–0.08 ng/ml) of sphingosine in samples. Black, standard data points; Red, sample data points. **(E)** *SMOX* was knocked down using shRNA targeting human SMOX at positions 574-594 in SK-Hep-1 cells. qRT-PCR was performed to verify knockdown of *SMOX* expression. **(F)** *SMOX*-depleted SK-Hep-1 cells were treated with sorafenib or spermine, and the cell viability of shCtrl and shSMOX was measured using viability assay. **(G)** *SPHK1* was knocked down using shRNA targeting *SPHK1* at positions 1126–1146 in SK-Hep-1 cells. qRT-PCR was performed to verify knockdown efficiency. **(H)** *SPHK1*-depleted SK-Hep-1 cells were treated with sorafenib or sphingosine, and the cell viability of shCtrl and shSPHK1 was measured by viability assay. **(I)** *CERS1* was depleted using shRNA targeting human *CERS1* at positions 526–546 in SK-Hep-1 cells. qRT-PCR was performed to verify knockdown knockdown of *CERS1* expression. **(J)** CERS1-depleted SK-Hep-1 cells were treated with sorafenib or sphingosine, and the cell viability of shCtrl and shCERS1 was evaluated by viability assay. *$p < 0.05$; **$p < 0.01$; **$p < 0.001$. # $p < 0.05$; ## $p < 0.01$. Data are presented as mean ± SD.

Supplementary Figure S3

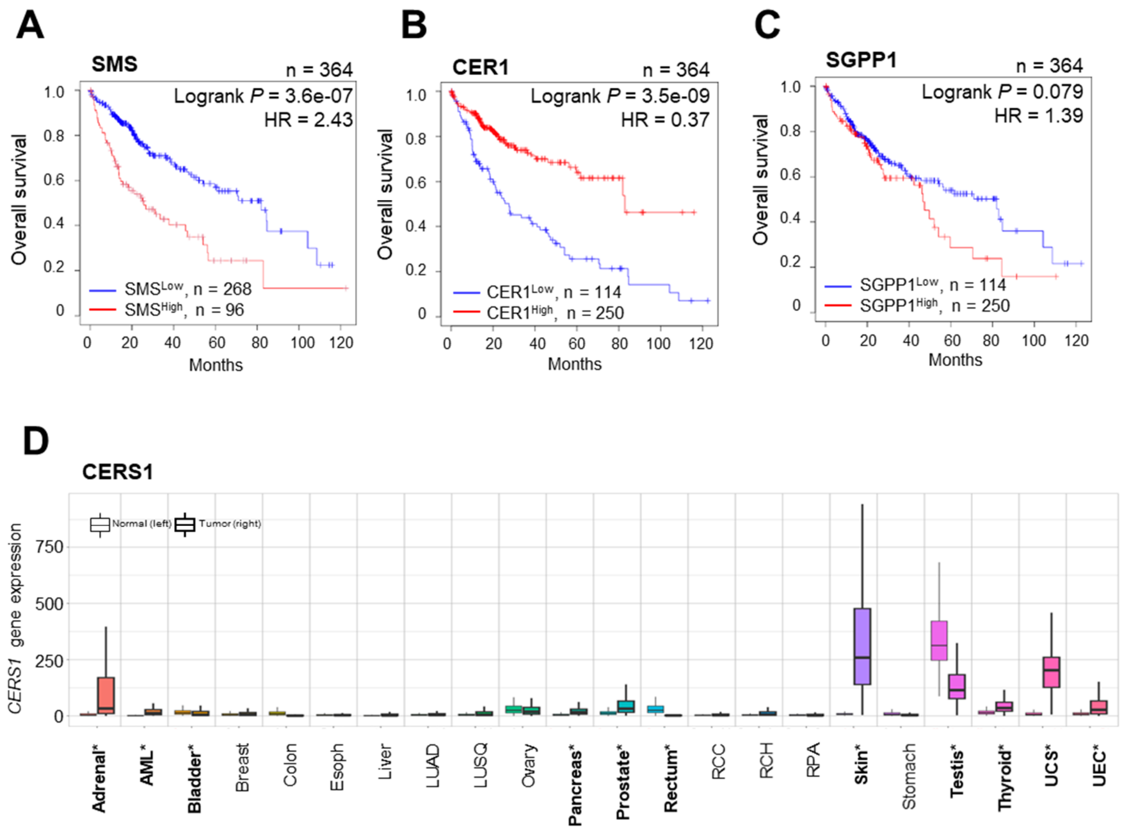

Figure S3. The survival rates of liver cancer patients according to the expression of the spermine synthase (SMS), ceramidase (CER1), and sphingosine-1-phosphate phosphatase 1 (SGPP1) synthetic enzymes. (A-C) The overall survival rates of liver cancer patients in TCGA and their expression levels of the spermine synthetic enzyme *SMS* **(A)**, ceramidase *CER1* **(B)**, and sphingosine-1-phosphate phosphatase 1 *SGPP1* **(C)** plotted using KM Plotter. **(D)** The relative gene expression of *CERS1* in normal (left) and tumor (right) tissues from adrenal cancer (Adrenal), acute myeloid leukemia (AML), bladder cancer (Bladder), breast cancer (Breast), colon cancer (Colon), esophageal cancer (Esoph), liver cancer (Liver), lung adenocarcinoma (LUAD), lung squamous cell carcinoma (LUSQ), ovary cancer (Ovary), pancreatic cancer (Pancreas), prostate cancer (Prostate), rectal cancer (Rectum), renal clear cell cancer (RCC), renal CH (RCH), renal PA (RPA), skin cancer (Skin), stomach

cancer (Stomach), testis cancer (Testis), thyroid cancer (Thyroid), uterine CS cancer (UCS), and uterine EC (UEC). *: Mann-Whitney $p<0.05$ and expression >10 in tumor or normal tissue.

Supplementary Figure S4

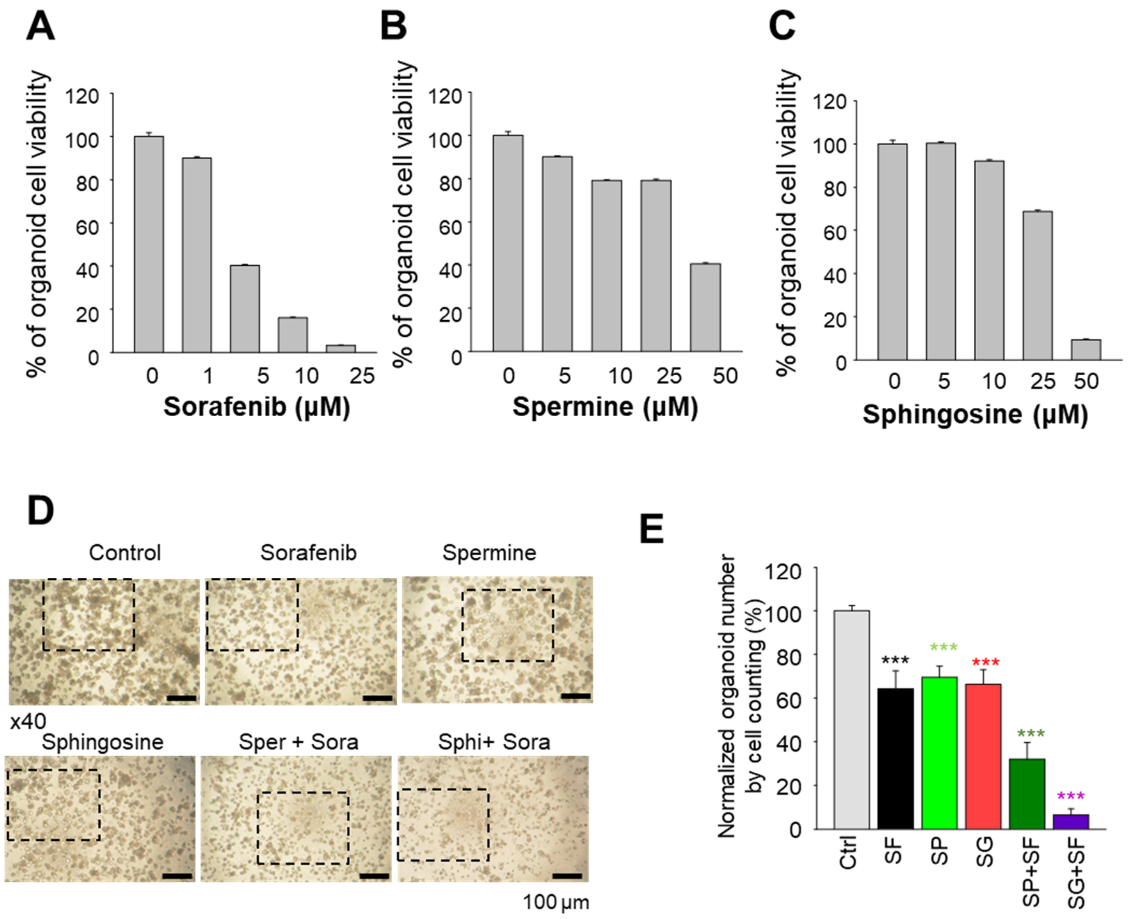

Figure S4. Evaluation of HCC organoid viability following treatment with sorafenib, spermine, and/or sphingosine in HCC organoids. (A-C) Organoids were exposed to increasing concentrations of sorafenib **(A)**, spermine **(B)**, or sphingosine **(C)** for 6 days and organoids cell viability was measured using CellTiter-Glo3D. **(D)** Representative enlarged images of HCC organoids shown in Figure 5E after treatment with sorafenib, spermine, and/or sphingosine. Scale bar, 100 μm. (**E)** Organoid formation efficiency and cell viability were assessed by cell counting method. The relative organoid cell viability was plotted. Data are presented as the mean ± SD. ***$p < 0.001$.

Supplementary Figure S5

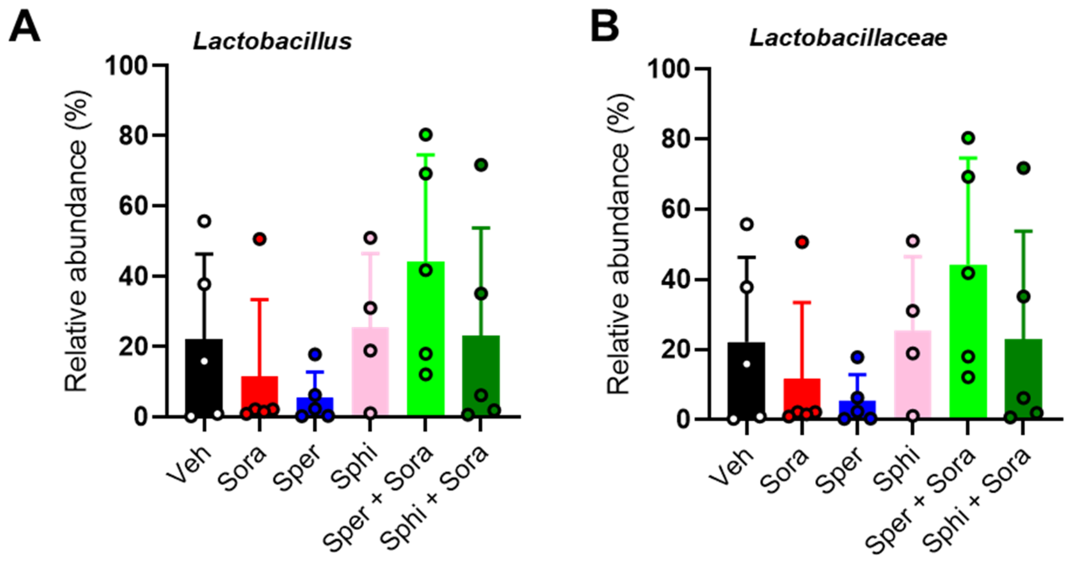

Figure S5. Relative abundance of *Lactobacillus* and *Lactobacillaceae* in gut microbiome analysis. Cecal samples were collected from mouse intestines, and DNA was extracted. The bacterial 16S rRNA gene (V4 region) was amplified, and the microbiome sequencing data were analyzed. Relative abundance of **(A)** *Lactobacillus* and **(B)** *Lactobacillaceae* in those cecal samples (n = 5 per group).

Supplementary Figure S6

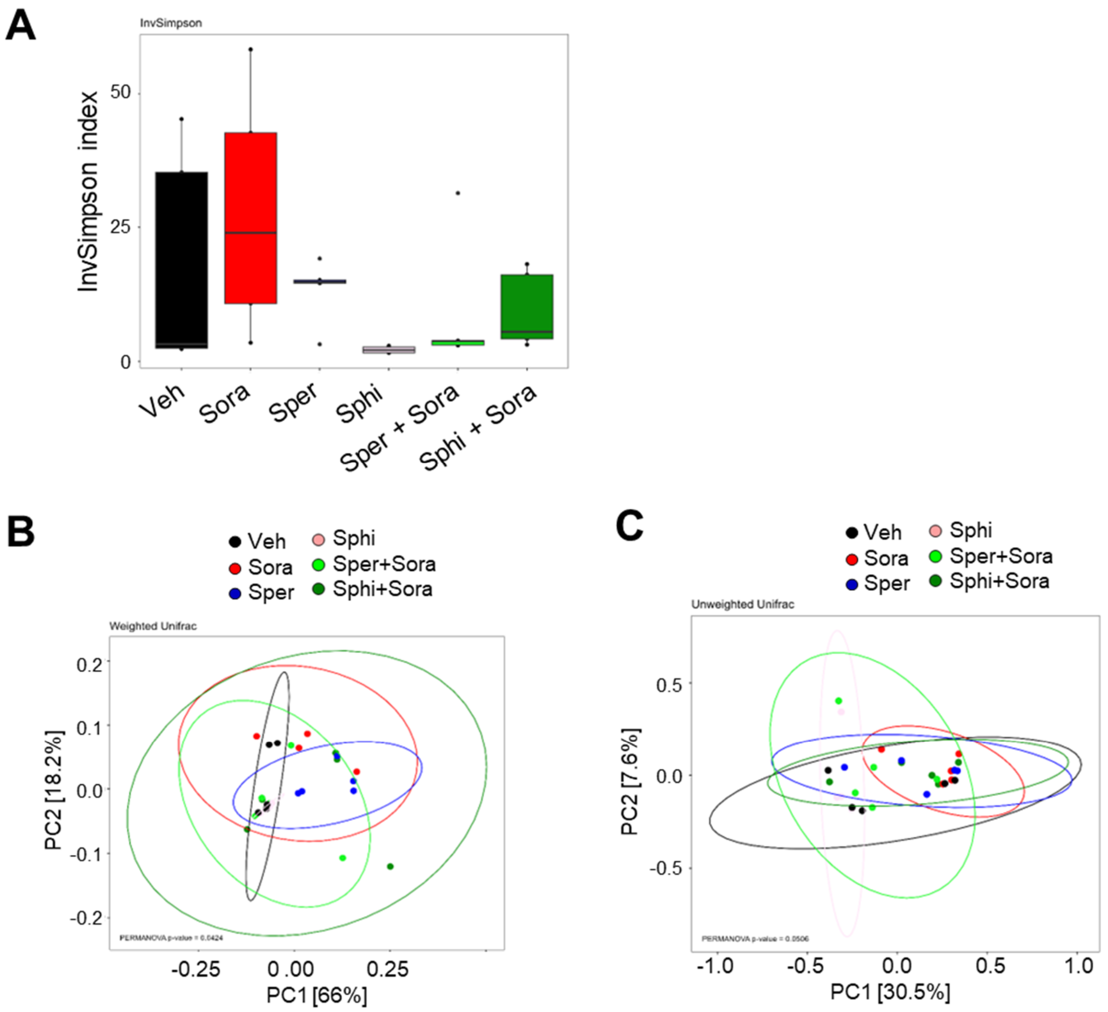

Figure S6. Gut microbiome analysis in HCC xenograft mice treated with sorafenib, spermine, and/or sphingosine. (A) Alpha diversity was analyzed using the Inverse Simpson index (n = 5 per group). **(B–C)** Beta diversity was analyzed using the weighted UniFrac **(B)** and unweighted UniFrac **(C)** distances.

Supplementary Table S1. List of 220 microbiome-derived metabolites

No.#	Microbial metabolites
1	N-Methylnicotinamide
2	Spermine
3	N-Acetyl-L-tryptophan
4	(E)-m-Coumaric acid
5	4-Aminobenzoic acid
6	Stearic acid
7	Phloretin
8	Thymidine
9	L-Lysine
10	2'-Deoxycytidine
11	Dimethyl trisulfide
12	Pimelic acid
13	Desaminotyrosine
14	Syringic acid
15	5-Hydroxymethyl-2-furancarboxylic acid
16	Glycolic acid
17	O-Desmethylangolensin
18	Hypoxanthine
19	Glycoursodeoxycholic acid
20	Methyl 2-(1H-indol-3-yl)acetate
21	Uridine 5'-monophosphate
22	N-Acetylornithine
23	Phenylacetylglycine
24	Folic acid
25	Taurocholic acid (sodium salt hydrate)
26	D-Mannitol
27	Thiamine (hydrochloride)
28	Glutaric acid
29	L-Ornithine
30	Hydroxyphenyllactic acid
31	Cytosine
32	3-Hydroxyhippuric acid
33	DL-3-Phenyllactic acid
34	L-Lactic acid
35	Acetic acid (magnesium tetrahydrate)
36	Allyl methyl sulfide
37	2,3-Butanediol
38	(R)-3-Hydroxybutanoic acid
39	Indole
40	Adenine
41	Glycochenodeoxycholic acid
42	Urea
43	Paraxanthine
44	Glycine
45	L-Ascorbic acid
46	Vanillic acid
47	Menaquinone-4
48	N-Acetylputrescine hydrochloride
49	Indole-3-pyruvic acid
50	Spermidine

No.#	Microbial metabolites
51	2-Methylpentanedioic acid
52	L-Phenylalanine
53	Cinnamoylglycine
54	trans-Cinnamic acid
55	Tryptophol
56	Glycocholic acid
57	Niacin
58	Pyruvic acid
59	cis,cis-Muconic acid
60	4-Hydroxyphenylacetic acid
61	Taurochenodeoxycholic acid
62	3-Methyl-2-oxobutanoic acid
63	Xanthine
64	Sodium 2-oxopropanoate
65	Oxalic Acid
66	Deoxycholic acid sodium salt
67	Tricarballylic acid
68	5-Hydroxyindole-3-acetic acid
69	6-Hydroxynicotinic acid
70	trans-3-Indoleacrylic acid
71	(R)-3-Hydroxybutanoic acid (sodium)
72	Phenylacetylglutamine
73	Dihydrouracil
74	N-(5-Aminopentyl)acetamide
75	D-Ribose(mixture of isomers)
76	5-Hydroxyindole
77	Phenyl acetate
78	Spermine (tetrahydrochloride)
79	Sodium Salicylate
80	Protocatechuic acid
81	Taurocholic acid (sodium)
82	Melilotic acid
83	L-Dihydroorotic acid
84	p-Cresyl sulfate (potassium)
85	Phosphorylethanolamine
86	L-Ornithine (hydrochloride)
87	γ-Aminobutyric acid
88	Cytidine 5'-monophosphate
89	L-Arginine (hydrochloride)
90	Inosinic acid (disodium)(hydrate)(1:2:X)
91	N-Methylsarcosine
92	Dimethyl sulfone
93	Thiamine monochloride
94	Ribitol
95	L-Lysine hydrochloride
96	Homovanillic acid
97	Norepinephrine (hydrochloride)
98	L-Serine
99	2,6-Diaminoheptanedioic acid
100	2'-Deoxycytidine-5'-monophosphoric acid

No.#	Microbial metabolites
**101**	Hydrocinnamic acid
**102**	DL-Glutamine
**103**	L-Aspartic acid
**104**	L-Threonine
**105**	DPPC
**106**	Hydroxytyrosol
**107**	L-Glutamic acid
**108**	L-Tyrosine
**109**	Lecithin
**110**	Cyclic AMP
**111**	Vitamin K
**112**	H-D-Trp-OH
**113**	4-Hydroxybenzoic acid
**114**	Pyrogallol
**115**	2-Phenylpropionic acid
**116**	Vitamin B12
**117**	Skatole
**118**	Glycochenodeoxycholic acid (sodium salt)
**119**	3-(3-Hydroxyphenyl)propionic acid
**120**	α-Hydroxyglutaric acid (disodium)
**121**	D-erythro-Sphingosine
**122**	Glycodeoxycholic acid (monohydrate)
**123**	Uridine 5′-diphosphoglucose (disodium salt)
**124**	3-Hydroxybenzoic acid
**125**	Imidazoleacetic acid (hydrochloride)
**126**	Taurochenodeoxycholic acid (sodium)
**127**	Hyodeoxycholic acid
**128**	Creatinine
**129**	Cyclic N-Acetyl-D-mannosamine
**130**	2,5-Dihydroxybenzoic acid
**131**	3-Indoleacetic acid
**132**	Lithocholic acid
**133**	2'-Deoxyuridine
**134**	Taurodeoxycholic acid
**135**	3,4-Dihydroxybenzeneacetic acid
**136**	Creatine
**137**	L-Asparagine
**138**	Pyridoxine
**139**	Pipecolic acid
**140**	Rhamnose
**141**	Biotin
**142**	Xanthosine
**143**	Salicyluric acid
**144**	trans-trans-Muconic acid
**145**	5'-Methylthioadenosine
**146**	Melatonin
**147**	Gallic acid (hydrate)
**148**	Adenosine monophosphate
**149**	Pyridoxine (hydrochloride)
**150**	D-Arabitol

No.#	Microbial metabolites
**151**	Adenosine
**152**	2-Hydroxyhexanoic acid
**153**	N-Acetyl-D-glucosamine
**154**	Trimethylamine N-oxide
**155**	Isovaleric acid
**156**	Allantoin
**157**	Hippuric acid
**158**	L-Tryptophan
**159**	L-Tartaric acid
**160**	Succinic acid
**161**	Indole-3-butyric acid
**162**	Vanillylmandelic acid
**163**	N-Acetyl-L-glutamic acid
**164**	3-Hydroxyphenylacetic acid
**165**	Thiamine nitrate
**166**	Salicylic acid
**167**	Rhamnose (monohydrate)
**168**	Glycodeoxycholic Acid
**169**	Dihydrocaffeic acid
**170**	L-Leucine
**171**	Homogentisic acid
**172**	4-Hydroxyphenylpyruvic acid
**173**	4-Pyridoxic acid
**174**	Taurodeoxycholic acid (sodium hydrate)
**175**	2'-Deoxyadenosine-5'-monophosphate
**176**	Flavin adenine dinucleotide (disodium salt)
**177**	5-Methyluridine
**178**	Riboflavin
**179**	Cholesterol
**180**	Gallic acid
**181**	Taurocholic acid
**182**	Phenylpyruvic acid
**183**	Quinolinic acid
**184**	3-Indolepropionic acid
**185**	D-(+)-Trehalose dihydrate
**186**	Deoxycholic acid
**187**	Nonadecanoic acid
**188**	N-Methylhydantoin
**189**	(S)-Leucic acid
**190**	Tartaric acid (disodium dihydrate)
**191**	(2-Aminoethyl)phosphonic acid
**192**	Inosinic acid
**193**	D-Galacturonic acid (hydrate)
**194**	L-Gulose
**195**	Benzoic acid
**196**	Tyrosol
**197**	L-Leucyl-L-alanine
**198**	L-Histidine
**199**	2-Amino-5-ureidopentanoic acid
**200**	L-Proline

No.#	Microbial metabolites
**201**	(S)-3,4-Dihydroxybutyric acid (lithium hydrate)
**202**	Disodium succinate
**203**	D-(-)-Lactic acid (sodium)
**204**	Thymidine-5'-monophosphate (disodium) salt
**205**	D-(+)-Trehalose
**206**	L-Valine
**207**	NSC 16590
**208**	3-Amino-2-methylpropanoic acid
**209**	D-Alanine
**210**	N-Acetylneuraminic acid
**211**	2,5-Furandicarboxylic acid
**212**	L-Cysteine
**213**	Trimethylamine N-oxide (dihydrate)
**214**	L-Methionine
**215**	Spermidine (hydrochloride)
**216**	Sodium 3-methyl-2-oxobutanoate
**217**	L-Ascorbic acid (sodium salt)
**218**	(-)-Aspartic acid
**219**	Aminomalonic acid
**220**	5-Aminovaleric acid

Supplementary Table S2. List of primers used for qRT-PCR

Target Gene	Primer	Sequences
Human *SMS*	Forward	5'- GTTCCAATCTCCACGTCTCC -3'
	Reverse	5'- TGAAGGGACACAGACGATCT -3'
Human *CER1*	Forward	5'- GAGCAACTTCCAGTACTCGG -3'
	Reverse	5'- AAGAGGACCCAGACAACGTA -3'
Human *SGPP1*	Forward	5'- AGGAAGTGGTGCTGGAATTG -3'
	Reverse	5'- GCAGGCTAAAGGAATGGTGA -3'
Human *SMOX*	Forward	5'- TGCAACAGCCTACAGTTTGT -3'
	Reverse	5'- CACACTTCTCCATGACGAGG -3'
Human *CERS1*	Forward	5'- TGTTTAACTACGCGGGATGG -3'
	Reverse	5'- GCAGCTGTAGAACTCCCATC -3'
Human *SPHK1*	Forward	5'- TGAAGACCTCCTGACCAACT -3'
	Reverse	5'- GACGCCGATACTTCTCACTC -3'
Human *GAPDH*	Forward	5'- TAAAGGGCATCCTGGGCTACACT -3'
	Reverse	5'- TTACTCCTTGGAGGCCATGTAGG -3'

Supplementary Table S3. GI_{20}, GI_{30}, and GI_{50} values of sorafenib, spermine, and sphingosine in SK-Hep-1 cells depleting SMOX, SPHK1, and CERS1, corresponding to Supplementary Figure S2F, S2H, and S2J.

**Compound**	**Sorafenib** (μM)			**Spermine** (μM)			**Sphingosine** (μM)		
	**GI_{20}**	**GI_{30}**	**GI_{50}**	**GI_{20}**	**GI_{20}**	**GI_{50}**	**GI_{20}**	**GI_{30}**	**GI_{50}**
**SK-Hep-1^{shCtrl}**	0.46	0.67	1.44	1.33	2.53	9.17	1.92	2.9	6.6
**SK-Hep-1^{shSMOX}**	0.23	0.37	0.93	0.57	1.24	5.88	-	-	-
**SK-Hep-1^{shSPHK1}**	0.17	0.30	0.88	-	-	-	0.99	1.57	3.94
**SK-Hep-1^{shCERS1}**	0.13	0.22	0.63	-	-	-	0.55	1.09	4.26

Table S4. Cox regression analysis for the survival of liver cancer patients expressing SMOX, SPHK1, and CERS1 in the KM plots used in Figure 5 and Supplementary Figure S3A-C.

**Patients**	**Endpoint**	**Gene expression**	**Number of patients (n)**	**Hazard ratio (HR)**	**95% (CI)**
HCC	Overall Survival n=364	SMS^{High}	96	2.43	1.71-3.45
		SMS^{Low}	268		
	Overall Survival n=364	$CER1^{High}$	250	0.37	0.26-0.52
		$CER1^{Low}$	114		
	Overall Survival n=364	$SGPP1^{High}$	100	1.39	0.96-2.01
		$SGPP1^{Low}$	264		
	Overall Survival n=364	$SMOX^{High}$	118	2.06	1.45-2.92
		$SMOX^{Low}$	246		
	Overall Survival n=364	$SPHK1^{High}$	222	1.54	1.07-2.22
		$SPHK1^{Low}$	142		
	Overall Survival n=364	$LASS1^{High}$	172	1.33	0.93-1.88
		$LASS1^{Low}$	192		
